# Stem cell-derived extracellular vesicles as immunomodulators: a novel paradigm for post-myocardial infarction repair and regeneration

**DOI:** 10.3389/fphar.2026.1860228

**Published:** 2026-06-10

**Authors:** Ali Raza, Panpan Wang, Fengjie Liu, Shumaila Arshad, Mulazim Hussain Asim, Zhenjiang Yang, Yu Cai

**Affiliations:** 1 College of Pharmacy, University of Sargodha, University Road Sargodha, Sargodha, Punjab, Pakistan; 2 The First Affiliated Hospital of Jinan University, Guangzhou, Guangdong, China; 3 State Key Laboratory of Bioactive Molecules and Druggability Assessment/International Cooperative Laboratory of Traditional Chinese Medicine Modernization and Innovative Drug Development of Ministry of Education (MOE) of China/Guangdong Key Lab of Traditional Chinese Medicine Informatization/International Science and Technology Cooperation Base of Guangdong Province/School of Traditional Chinese Medicine, Jinan University, Guangzhou, Guangdong, China; 4 Department of Pharmacy, Superior University Sargodha Campus, Sargodha, Punjab, Pakistan; 5 Shenzhen Traditional Chinese Medicine Hospital, Shenzhen, Guangdong, China; 6 State Key Laboratory of Bioactive Molecules and Druggability Assessment, Jinan University/International Cooperative Laboratory of Traditional Chinese Medicine Modernization and Innovative Drug Development of Ministry of Education (MOE) of China/Guangdong Key Lab of Traditional Chinese Medicine Informatization/International Science and Technology Cooperation Base of Guangdong Province/School of Pharmacy, Jinan University, Guangzhou, Guangdong, China

**Keywords:** immunomodulators, macrophage polarization, myocardial infarction, regenerative medicine, stem cell-derived extracellular vesicles

## Abstract

Myocardial infarction (MI) initiates a rapid and highly coordinated immune response that is essential for the clearance of necrotic tissue and activation of reparative processes. However, prolonged or dysregulated post-MI inflammation can exacerbate myocardial injury, promote adverse cardiac remodeling, and ultimately contribute to heart failure. Although current therapeutic strategies improve survival and symptom management, they remain limited in their ability to restore lost cardiomyocytes or effectively modulate the post-infarction immune microenvironment. In this context, stem cell-derived extracellular vesicles (EVs) have emerged as promising cell-free therapeutic candidates due to their immunomodulatory, regenerative, and paracrine properties. These nanoscale vesicles carry a diverse cargo of bioactive molecules, including microRNAs, proteins, lipids, and other signaling mediators that regulate intercellular communication and tissue repair. EVs derived from mesenchymal stem cells, cardiac progenitor cells, and induced pluripotent stem cells have demonstrated the ability to modulate key immune pathways by attenuating neutrophil-mediated inflammatory injury, promoting macrophage polarization towards a reparative M2 phenotype, and regulating T-cell responses by suppressing pro-inflammatory activity while enhancing regulatory T-cell function. Collectively, these effects help restore immune homeostasis and reduce adverse cardiac remodeling following MI. Moreover, advances in EVs engineering, cargo modification, and targeted delivery systems may enhance their therapeutic efficacy and translational potential. However, several critical challenges, including large-scale production, cargo heterogeneity, and the lack of standardized protocols for isolation and characterization, still need to be addressed before successful clinical translation. This review summarizes the current understanding of stem cell-derived EVs biology, comparative advantages over conventional and cell-based therapies, and their immunomodulatory mechanisms in post-MI repair. Moreover, it highlights recent innovations and the major challenges that must be addressed for successful clinical translation.

## Graphical Abstract

**Figure:**
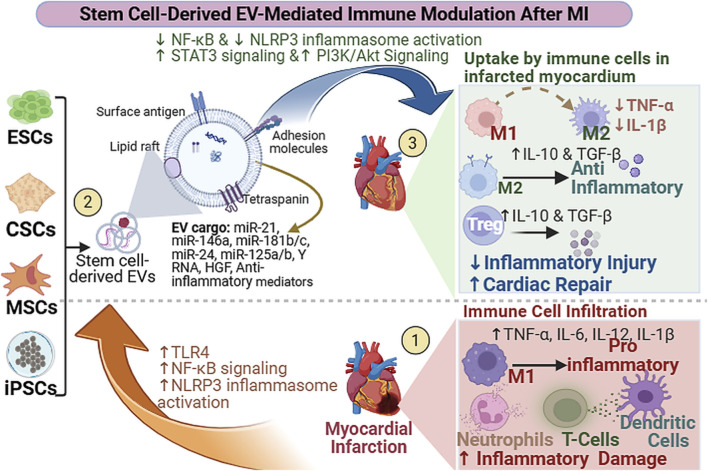

## 1 Introduction

Myocardial infarction (MI) is still a major cause of illness and death worldwide, mainly because heart muscle cells are permanently lost and subsequently the heart undergoes harmful structural changes (Rohani et al., 2022). Following MI, necrotic cardiomyocytes release damage-associated molecular patterns (DAMPs) that trigger a sterile inflammatory response and activate the innate immune system, leading to rapid recruitment of neutrophils and macrophages to the injured myocardium. Subsequently, adaptive immune cells, particularly T lymphocytes, contribute to the regulation of inflammation, removal of cellular debris, and tissue repair through cytokine-mediated signaling. However, excessive or prolonged immune activation may exacerbate myocardial injury, promote fibrosis, impair cardiac function, and ultimately lead to heart failure (Liu H. et al., 2016; Ong et al., 2018). Thus, fine-tuning the post-MI immune response has emerged as a critical therapeutic target.

Current therapeutic strategies, including reperfusion techniques and pharmacological interventions, largely focus on restoring blood flow and alleviating symptoms but fail to regenerate lost myocardium or adequately control post-infarction inflammation (Lazar et al., 2018; Frangogiannis, 2014). In this context, stem cell-based therapies have gained considerable attention due to their regenerative and immunomodulatory potential (Linke et al., 2005). Studies involving autologous bone marrow cells (Leistner et al., 2011; Wollert et al., 2004) and cardiac stem cells (CSCs) (Chugh et al., 2012) showed clear and sustained improvements in left ventricular ejection fraction and reduced scar/infarct size over several years. Interestingly, not all studies have reported consistent benefits of stem cell therapy. The intracoronary delivery of mesenchymal stem cells (MSCs) in both porcine models and humans with acute MI (AMI) resulted in a significant reduction in absolute myocardial blood flow, poor retention of transplanted cells in the infarcted area, and no improvement in global cardiac function (Gyöngyösi et al., 2011; Gyöngyösi et al., 2015; Gyöngyösi et al., 2017). A major reason for this treatment failure is that resident or transplanted stem cells in the heart have a very short lifespan of a few days, which greatly restricts their ability to regenerate damaged myocardium (van Berlo et al., 2014). In addition, their clinical application is limited by challenges such as poor engraftment, risk of immune rejection, arrhythmogenicity, and ethical concerns (Madonna et al., 2016).

These limitations have shifted attention towards the paracrine mechanisms through which stem cells exert their benefits. Early work by Chen et al. (2010) and others demonstrated that MSC secretions contain a wide array of proteins, lipids, and RNA species that can be taken up by recipient cardiomyocytes, such as H9C2 cells, and modulate their function (Chen et al., 2010; Collino et al., 2010; Tomasoni et al., 2013). Similarly, several findings suggest that the regenerative and immunomodulatory effects of MSCs are due to the secretion of paracrine factors, such as extracellular vesicles (EVs) (Caplan and Dennis, 2006; Lai et al., 2010; Timmers et al., 2011). EVs are small, membrane-bound particles released by nearly all types of cells. They act as messengers by carrying proteins, microRNAs (miRs), messenger RNA (mRNA), and lipids from one cell to another and thus play a crucial role in intercellular communication (Ibrahim and Khan, 2025; Maas et al., 2017; Kalluri and LeBleu, 2020).

Moreover, EVs released by different cells can influence the activity of other cells, which can stimulate major biological reactions (Tomasoni et al., 2013; Borges et al., 2013; Bruno et al., 2012; Bruno et al., 2009). Several clinical trials have shown that stem cell-derived EVs (stem cell-EVs) are safe and may offer therapeutic benefits in various conditions (Lightner et al., 2023; Zhu Y.-G. et al., 2022; Fu et al., 2023; Van Delen et al., 2024). Supporting this evidence, stem cell-EVs have demonstrated potent immunomodulatory effects across multiple pathological conditions, including autoimmune disorders, inflammatory diseases, tissue injury, and regenerative medicine, where they regulate macrophage polarization, dendritic cell activation, and T-cell responses (Arsl et al., 2013; Zhao et al., 2015; Davidson et al., 2017). These findings provide a strong rationale for exploring their therapeutic potential in post-MI immune dysregulation. In the context of MI, stem cell-EVs have emerged as promising immunomodulatory agents for post-MI repair due to their ability to regulate multiple immune and reparative pathways. These EVs suppress excessive neutrophil infiltration, promote macrophage polarization towards the anti-inflammatory M2 phenotype, and modulate T-cell-mediated inflammatory responses, thereby reducing myocardial inflammation and fibrosis. Overall, EVs exert cardioprotective effects by modulating inflammation and promoting tissue repair following MI.

This review focuses initially on EVs biology and their advantages over different conventional therapies, then discusses the immune system involvement, particularly neutrophils, macrophages, and T cells, in myocardial infarction reperfusion injury (MIRI). Finally, we provided the immunomodulatory and anti-inflammatory role of stem cell-EVs in post-MI cardiomyocytes repair, as well as the recent innovations and challenges to be addressed for clinical trials.

## 2 Biology and advantages of EVs

**FIGURE 1:**
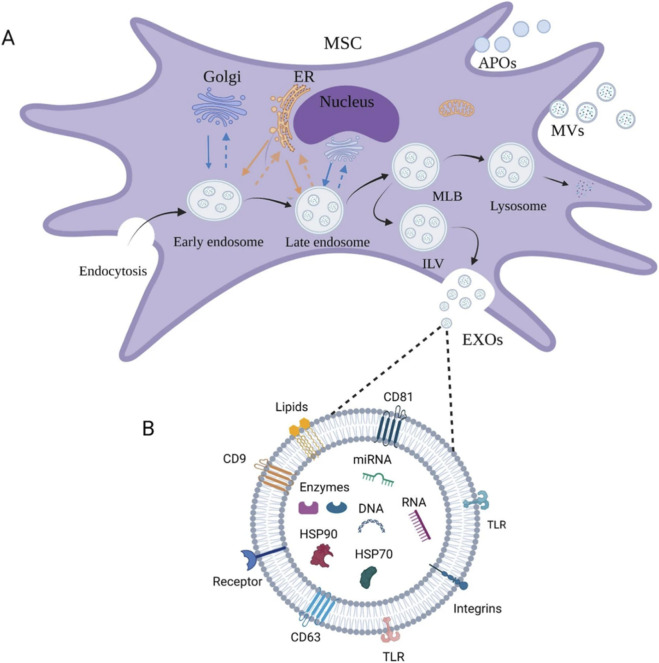
**(A)** The figure illustrates how mesenchymal stem cells (MSCs) produce different types of extracellular vesicles (EVs). After endocytosis, early endosomes progress to late endosomes, where small intraluminal vesicles (ILVs) form inside multivesicular bodies (MVBs). These MVBs have two possible fates: they may merge with lysosomes and be broken down, or they may fuse with the cell membrane and release their ILVs as exosomes. Other EV subtypes arise by different mechanisms, for example, microvesicles bud outward directly from the plasma membrane, while apoptotic bodies are released when cells undergo programmed death. **(B)** The second panel summarizes the structural features and common contents of MSC-derived exosomes. The figure is reproduced from Kou M., Huang L., Yang J., et al. Cell Death and Disease (2022), licensed under CC BY 4.0. (Kou et al., 2022).

Based on their size, origin, and biogenesis, EVs are broadly classified into exosomes (30–150 nm), microvesicles (100–1000 nm), and apoptotic bodies (>1000 nm) (Blackwell and Stanford, 2023; Yáñez-Mó et al., 2015). The biogenesis of exosomes begins within the endosomal pathway (Lai et al., 2010; Doyle and Wang, 2019; Heijnen et al., 1999), as shown in Figure 1. Exosomes carry diverse bioactive molecules reflecting parent cell status and can transfer G protein-coupled receptors (GPCRs), thereby modify receptor distribution and signal in recipient cells (Estelles et al., 2007; Pironti et al., 2015; Yellon and Davidson, 2014). Exosome formation is mainly controlled by the ESCRT (endosomal sorting complex required for transport) machinery, which helps sort cargo and shape and release vesicles. However, ESCRT-independent pathways involving lipids like ceramide and proteins such as CD9, CD63, and CD81 also play a role (Colombo et al., 2014; Stuffers et al., 2009). Microvesicles are formed when the cell membrane directly buds outward and pinches off. This process is controlled by changes in the cell’s cytoskeleton and the movement of membrane lipids, especially phosphatidylserine to the outer surface (Yáñez-Mó et al., 2015). On the large size scale are the apoptotic bodies are emitted because of the cellular disassembly of the cell in programmed cell death. The vesicles hold various remnants inside the cell, such as fragments of the nucleus, organelles, and proteins (histones), and they are all wrapped when the cell dies (Chen et al., 2024a; Zhu et al., 2025). An extensive variety of stem cell populations has been discovered as valuable sources of exosomes possessing cardioprotective and regenerative capabilities. These include MSCs, CSCs, cardiosphere-derived cells (CDCs), and induced pluripotent stem cells (iPSCs).

Among the different EV subtypes, exosomes are the most widely studied in MI because of their small size, high stability, efficient tissue penetration, and capacity to deliver cardioprotective bioactive molecules involved in regulating inflammation and tissue repair. In contrast, microvesicles also participate in intercellular signaling but face translational challenges because of their larger size, heterogeneity, and limited standardization. Apoptotic bodies have been less explored in cardiac regeneration and are mainly associated with the clearance of dying cells and inflammatory signaling. Overall, current evidence indicates that exosomes represent the most promising EV subtype for immunomodulation and cardiac repair following MI (Kalluri and LeBleu, 2020).

Stem cell-EVs offer significant clinical advantages due to their biocompatibility, low immunogenicity, stability, and ability to cross biological barriers while preserving cargo integrity (Kou et al., 2022; Li et al., 2022; de Abreu et al., 2020). They enable efficient intercellular communication, are small, scalable, and safer than whole-cell therapies as they lack proliferative or malignant potential (Kou et al., 2022; Pegtel et al., 2010; Vrijsen et al., 2010; Shao et al., 2017; Pezzana et al., 2021). Their natural targeting ability, engineering flexibility, and capacity for drug or RNA delivery, including inhaling applications, make them superior to traditional nanocarriers and highly promising for diverse therapeutic uses (Kou et al., 2022; de Abreu et al., 2020; Hassanzadeh et al., 2021; Takakura et al., 2024; Song et al., 2019; Alvarez-Erviti et al., 2011; Popowski et al., 2022). However, EVs are diverse in size, content, and function, even within the same type. This variation depends on the parent cell and its conditions, which can influence their therapeutic effects. Therefore, controlling EV heterogeneity is important for reliable clinical use (de Abreu et al., 2020; Forsberg et al., 2020; Menard et al., 2013).

## 3 Immune cell dynamics in Post-MI inflammation and repair

AMI leads to sterile injury and cell destruction in the heart by initiating a complex immune response. The post-MI repair process follows three successive steps, involving an inflammatory step to remove debris, a reparative/anti-inflammatory step to repair tissue, and a remodeling stage for scar formation (Anzai et al., 2012; Feng et al., 2023; Liu et al., 2024; Marinković et al., 2019). The innate as well as the adaptive immune responses are essential in the regulation of these stages. A great increase in the activity of Caspase-3/7 was detected when cardiomyocytes were exposed to a hypoxic period (24 h), hence leading to increased apoptosis and cell death (Yu et al., 2013a; Yu et al., 2013b). Death of cardiomyocytes in MI triggers an immune response through the release of chemical signals (DAMPs), which stimulate a non-specific (innate) immune response and subsequently a specific (adaptive) immune response (Feng et al., 2023; Gupta and Knowlton, 2007; Liu J. et al., 2016). Secondly, reperfusion following MI can rather perversely trigger secondary MIRI, largely due to overproduction of ROS and exacerbated inflammation, which in the end can lead to a further worsening of heart activity and the development of arterial heart failure (Hou et al., 2022; Chen et al., 2017).

Neutrophils constitute one of the earliest immune cells to infiltrate the injured heart. Profiling of gene expression of cardiac neutrophils 24 h after MI reveals that the pro-inflammatory N1 signature genes (including IL-1β, TNF-α, and S100A8/A9) peak within the first day, whilst anti-inflammatory N2-related genes (for example, IL-10, TGF-β, and arginase-1) increase after 5–7 days (Ma et al., 2016; Puhl and Steffens, 2019). Unregulated or excessive neutrophil recruitment exacerbates MIRI through the release of ROS, proteases, matrix metalloproteinases (MMPs), and NETs (neutrophil extracellular traps), which lead to infarct wall thinning and persistent inflammation (Ma et al., 2016; Ma, 2021; Zhang et al., 2022; Zhang X. et al., 2018). Excessive neutrophil infiltration contributes to secondary myocardial injury, making neutrophil-driven inflammation an important therapeutic target for stem cell-EV-based interventions. The cytokine release also assists the monocytes’ selection for M1 macrophages, enhancing tissue destruction (Kim et al., 2024).

Resident cardiac macrophages (CCR2^-^, MHC-II^low/high^) are critical for early protection by performing efferocytosis, modulating inflammation, and supporting electrical conduction and angiogenesis (Bajpai et al., 2019; Dick et al., 2019). Nevertheless, in the case of severe ischemic injury, these defensive macrophage subsets are quickly exhausted and substituted by pro-inflammatory Ly-6C^high^ CCR2^+^ monocyte-derived macrophages, which amplify the inflammation and matrix remodeling (Chen et al., 2024b; DeBerge et al., 2020; Sager et al., 2016). Nahrendorf et al., (2007) detailed two sequential phases of involvement of monocytes that occurred after MI. Initially, Ly-6C^high^ monocytes are predominant, and they play a phagocytic, proteolytic, and pro-inflammatory role via CCR2, and later they turn into Ly-6C^low^ monocytes and mediate angiogenesis, activation of myofibroblasts, collagen deposition, and VEGF (vascular endothelial growth factor) production via CX3CR1 signaling (DeBerge et al., 2020; Nahrendorf et al., 2007; Hilgendorf et al., 2014). The infiltrated macrophages are functional and highly varied: the early inflammatory response (days 1–3) is dominated by classically activated M1 macrophages, which initiate leukocyte recruitment, necrotic tissue clearance, enhanced inflammatory signaling, and mediate extracellular matrix destruction. Conversely, alternatively activated M2 macrophages emerge after day 5, inhibit inflammation, promote angiogenesis, trigger fibroblast activity, and stabilize scar tissue through the production of cytokines (IL-4/IL-13, IL-10, arginase-1, and TGF-β) (Ben-Mordechai et al., 2013; Fran et al., 2013; Leor et al., 2006; Yan et al., 2013). Because macrophage polarization strongly influences the balance between inflammation and repair, modulation of macrophage phenotype represents a major mechanism of stem cell-EV-mediated cardiac protection.

DAMPs, including HMGB1 (high mobility group box 1) and S100A8/A9, along with NETs, stimulate dendritic cells (DCs) through TLR4-based signaling and enhance adaptive immune responses (Xue et al., 2019). The exosomes of conventional DCs significantly enhance the expression of the pro-inflammatory cytokines IFN-γ and TNF-α, which cause activation and recruitment of macrophages, neutrophils, and CD4^+^ T cells, including Th-1 cells and Th-17 cells, against the cardiomyocytes as reported in C57BL/6 mice (Liu J. et al., 2016; Xue et al., 2019; Liu et al., 2018; Van der Borght et al., 2017; Zhu L.-P. et al., 2018). These immune cells and cytokines are involved in the induction of autoimmunity in the cardiomyocytes post-MI. In the later stages of MI, DCs exhibited decreased recruitment of pro-inflammatory Ly6C^high^ monocytes and M1 macrophages, whereas the infiltration of reparative Ly6C^low^ monocytes and M2 macrophages into the infarcted myocardium was markedly increased, along with reduced pro-inflammatory cytokines, including IL-1β, IL-18, and TNF-α (Anzai et al., 2012). The clinical and preclinical trials demonstrated that the treatment with tolerogenic DCs led to improved heart function and reduced infarct size by increasing FOXP3^+^ T regulatory (Treg) cells and M2 macrophages after MI (Choo et al., 2017; Nagai et al., 2014; Zhu et al., 2016). Hence, regulation of DC activation and antigen presentation may also contribute to the immunomodulatory effects of stem cell-derived EVs.

In mice following AMI, CD8^+^ T lymphocytes infiltrate the myocardium ischemic area at day 1 and release granzyme B, which induces cardiomyocyte apoptosis, adverse ventricular remodeling, and cardiac functional impairment (Santos-Zas et al., 2021). By day 7 post-MI, a specific population of CD8^+^ cells with AT2R expression (CD8^+^ AT2R^+^ T cells) accumulated in the infarcted myocardium of rats, which reacted to the stimulation of angiotensin II by secreting IL-10 and enhancing the anti-inflammatory effect on ischemic damage (Curato et al., 2010). Similar cardioprotective effects are also observed with CD4^+^ AT2R^+^ T cells after MI (Skorska et al., 2015). Among CD4^+^ T cells, Th-1 and Treg cells are the main subsets, while Th-2, Th-17, Th-22, and Th-9 cells remain less abundant in the post-MI period (Yan et al., 2013; Lin et al., 2013). Pathogenic CD4^+^ T cell release cytokines, including IL-21, which facilitate the apoptosis of reparative Ly-6C^low^ macrophages and increase the number of neutrophils and MMP-9 expression, while decreasing myofibroblast populations, and MMP-12 expression (Kubota et al., 2021; Wang K. et al., 2018). Similarly, Th-1/Th-17 cells inhibited cardiomyocyte proliferation and promoted their apoptosis, as reported in juvenile mice MI model (Li et al., 2020). The high number of immature neutrophils and monocytes in AMI patients is also associated with the high level of IFN-γ production by CD4^+^ T cells via a contact-independent mechanism, mediated by IL-12 (Fraccarollo et al., 2021). Conversely, protective FOXP3^+^ Treg cells decrease neutrophil, macrophage, and CD8^+^ T cells infiltration and inhibit TNF-α, IFN-γ, IL-17, and IL-1β and increase IL-10 and TGF-β expression (Blanco-Domínguez et al., 2022; Bönner et al., 2013; Borg et al., 2017; Matsumoto et al., 2011; Tang et al., 2012; Zhuang et al., 2022). Their activation inhibits Th-1 cells, supports M2 macrophage polarization and myofibroblast function, and reduces the risk of cardiac rupture (Hofmann et al., 2012; Li et al., 2019; Okoye et al., 2014; Sharir et al., 2014; Weirather et al., 2014). Th2-derived IL-2 expands Treg cells and suppresses Th-1/Th-17 responses, while Th22-derived IL-22 prevents ventricular dysfunction and heart failure (Tang et al., 2018; Zeng et al., 2016). Overall, T-cell-mediated inflammatory signaling represents another important immune pathway that may be therapeutically regulated by EV-based approaches.

Mature B cells play a more severe role in the injury due to the secretion of CCL7 early after MI and recruitment of CCR2-dependent Ly-6C^high^ monocytes, exacerbation of inflammation, and cardiac dysfunction (Sun et al., 2022; Zouggari et al., 2013). One dose of Rituximab, a monoclonal antibody that selectively targets human B cells, has shown clinical trial safety in acute ST-Elevation MI (STEMI) (Zhao et al., 2021). As an adaptation, however, in later stages, regulatory B cells (Breg cells) accumulate and provide cardioprotection by promoting the production of IL-10, neutrophil apoptosis, and M2-like macrophage polarization while reducing Ly6C^high^ monocyte infiltration, thus maintaining cardiac functionality (Huang et al., 2024; Jiao et al., 2021; Rocha-Resende et al., 2021; Wu et al., 2019; Xu et al., 2022). Immune cell populations are known to be regulated by stem cell-EVs during post-MI repair and regeneration, as discussed in the next section. Table 1 summarizes the temporal recruitment and functional polarization of innate and adaptive immune cells after MI. Collectively; these immune cell populations critically influence the balance between inflammatory injury and cardiac repair following MI. Given their central role in post-MI remodeling, these immune pathways represent important therapeutic targets for stem cell-EV-based interventions, which are discussed in the following section.

**TABLE 1:** Immune cell dynamics after MI and functional roles in repair.

Immune cell type	Early post-MI role	Late/Reparative role	Key cytokines/Signals	Impact on remodeling	Ref.
Neutrophils	Debris clearance, ROS release, NET formation	Efferocytosis, resolution of inflammation	IL-1β, TNF-α, ROS → IL-10, TGF-β (later)	Excess worsens cardiac injury and remodeling	Puhl and Steffens (2019)
Monocytes (Ly6Chigh)	Pro-inflammatory recruitment via CCR2	Differentiate into reparative macrophages	CCL2, TNF-α → VEGF, IL-10 (later)	Drive inflammation-to-repair transition	DeBerge et al. (2020)
Macrophages (M1/M2)	M1 dominates early, amplify inflammation	M2 promotes angiogenesis and fibrosis control	M1: IL-6, TNF-α; M2: IL-10, TGF-β	Central regulators of scar quality	Leor et al. (2006)
Dendritic Cells	Antigen presentation, adaptive activation	Tolerogenic DCs promote Treg cells	IL-12, IFN-γ → IL-10, TGF-β (later)	Modulate autoimmunity and fibrosis	Xue et al. (2019)
CD4^+^ T cells	Th-1/Th-17 amplify inflammation	Treg cells suppress inflammation	IFN-γ, IL-17 → IL-10, TGF-β (later)	Balance determines adverse vs. adaptive remodeling	Borg et al. (2017)
CD8^+^ T cells	Cytotoxic cardiomyocyte injury	Limited reparative role	Granzyme B, TNF-α	Excess worsens function	Santos-Zas et al. (2021)
B cells	Early CCL7-mediated monocyte recruitment	Regulatory B cells produce IL-10	CCL7 → IL-10 (later)	Biphasic: harmful early, protective later	(Jiao et al., 2021)

## 4 Immunomodulatory properties of stem cell-derived EVs in post-MI cardiac repair

**FIGURE 2:**
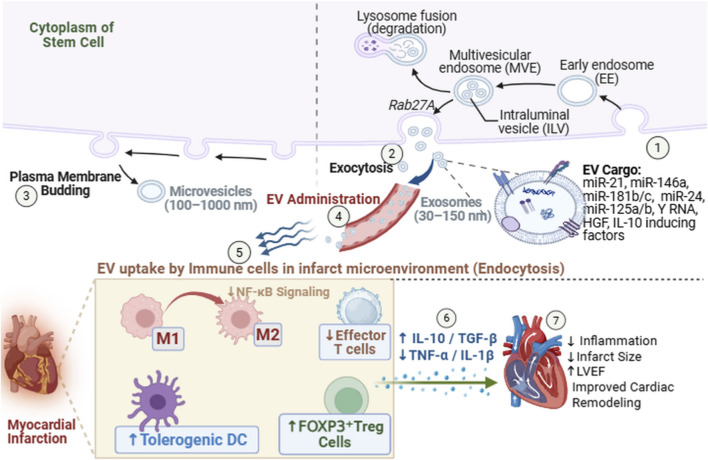
Immunomodulatory effects of stem cell-derived EVs in post-infarction cardiac repair. This diagram depicts the formation, release, therapeutic use, and immune-regulating effects of stem cell-derived EVs in the heart after myocardial infarction.

The stem cells have been widely researched for their immunoregulatory and anti-inflammatory properties in many diseases. MSC therapies and adult stem cell responses cause a regulated, temporary immune response to infarction, which recruits and reprograms macrophages to adopt reparative phenotypes, restrain fibrosis, and stabilizes the infarcted tissue (Vagnozzi et al., 2020; Zhang et al., 2020). Consistent with these findings, MSC therapy promotes M2 macrophage polarization and enhances the efferocytosis of apoptotic neutrophils, resulting in a significant reduction of MIRI and improved cardiac function in Sprague-Dawley rats. At the same time, the MSCs regulate adaptive immunity and inflammatory signaling, which decreases the immune cells’ infiltration, oxidative stress, and infarct size after MIRI (Chen et al., 2017; Azevedo et al., 2020). Notably, in addition to MSCs and CSCs, their conditioned media are also capable of significantly reducing post-MIRI inflammation (Djouad et al., 2007; Huang et al., 2011; Rafei et al., 2009). These findings pointed early on to a paracrine mechanism. Studies confirmed that much of this activity is carried out by EVs, especially exosomes, released by MSCs (Lai et al., 2010; Bian et al., 2014). Taken together, initial experiments indicated that MSCs have a wide ability to control innate and adaptive immunity (Aggarwal and Pittenger, 2005; Bartholomew et al., 2002; Chiossone et al., 2016; Corcione et al., 2006; Jiang et al., 2005), but recent evidence now clearly shows that EVs, especially exosomes, play a central role in such actions (Lai et al., 2010; Bian et al., 2014), as illustrated in Figure 2.

### 4.1 EVs from mesenchymal stem cells

Following MI, bone marrow MSCs (BMMSCs) respond to inflammatory cues such as TNF-α and local hypoxia by releasing a wide range of paracrine mediators that restore immune balance and limit excessive inflammation (Selvasandran et al., 2018). Additionally, specific miRs appear to be key players in these processes. Treatment with MSC-Exo decreased levels of miRs that harm cardiac function, such as miR-130, miR-378, and miR-34, alongside increased expression of miR-29 and miR-24, which are associated with improved myocardial remodeling (Shao et al., 2017).

**FIGURE 3:**
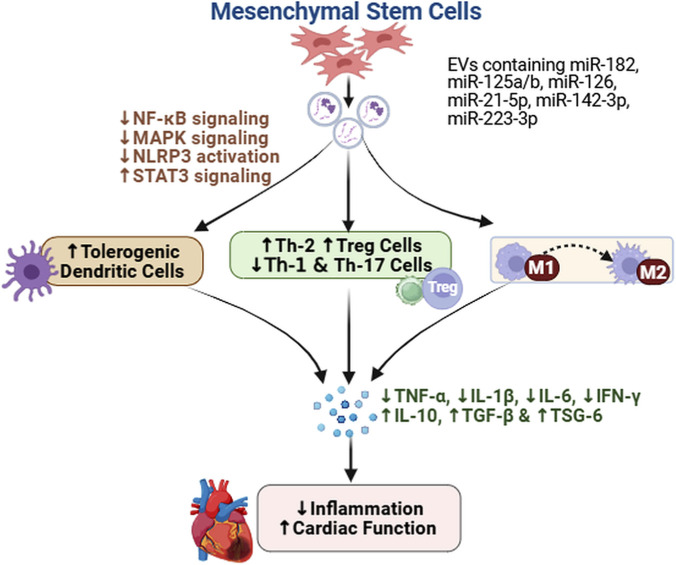
Immunomodulatory effects of mesenchymal stem cell (MSC)-derived EVs after MI.

MSC-Exo broadly modulates inflammatory pathways, primarily by shifting macrophages/monocytes from a pro-inflammatory M1 state towards an anti-inflammatory M2 phenotype in an inflammatory environment, both *in vivo* and *in vitro* studies (Li et al., 2022; Deng et al., 2019; Sun et al., 2018; Xu et al., 2019; Zhao et al., 2019), as shown in Figure 3. Several other studies support this pattern, which shows that MSC-Exo enriched with miR-182 reprogrammed macrophages via TLR4-dependent signaling pathways (Zhao et al., 2019). Likewise, MSC-EVs enriched with miR-125a/b enhances M2 macrophage polarization, decrease inflammatory cytokines (IL-1β, IL-6, TNF-α), stimulate angiogenesis, and suppress fibroblast activation in both mice and swine MI models (Chen et al., 2020; Gao et al., 2023). The cardioprotective effects of miR-125b are mediated through downregulation of SIRT7 (Sirtuin 7), whereas miR-125a-5p confers cardioprotection by targeting Klf13, Tgfbr1, and Daam1, which are regulators of apoptosis, fibrosis, and cardiac remodeling (Chen et al., 2020; Gao et al., 2023). These MSC-induced macrophages express elevated anti-inflammatory markers such as arginase-1, CD206, and secrete high levels of IL-10 and TSG-6 (tumor necrosis factor-stimulated gene-6), which not only suppress activated T-cell proliferation but also promote the expansion of FOXP3^+^ Treg cells (Heo et al., 2019). MSC-EVs can also regulate the behavior of regulatory macrophages (Mregs) by lowering IL-23 and IL-22 levels while increasing prostaglandin E_2_, which promotes a pro-resolving and anti-inflammatory phenotype (Hyvärinen et al., 2018). Collectively, MSC-EVs showed improved cardiac function, decreased inflammation, and enhanced cardiomyocyte regeneration (Gao et al., 2023).

BMMSCs and their EVs also modulate DCs, which adds another layer to their immunomodulatory profile. MSCs express TSG-6, which inhibits the activation of MAPK and NF-kB in bone marrow-originated DCs and stops their maturation (Liu et al., 2014). These effects are reflected by MSC-EVs via miRs, such as miR-21-5p, miR-142-3p, and miR-223-3p (Reis et al., 2018). DCs that have been trained on MSC-EVs express fewer maturation markers, i.e., CD83, CD86, CD40, and MHC II, and produce fewer IL-6 and IFN-γ and more IL-10/TGF-β (Reis et al., 2018; Shahir et al., 2020). These tolerogenic DCs lower the number of Th-17 cells and promote FOXP3^+^ Treg cells in their functions (Shahir et al., 2020; Favaro et al., 2016), which illustrates the wide-ranging and integrated effects of MSC-EVs on immunoregulation.

Beyond their effects on DCs, MSC-EVs also exert widespread immunomodulatory functions by directly influencing T-cell behavior and cytokine profiles. MSC-Exo therapy mitigated the severity of the disease, improved survival, and increased populations of human Treg cells in a mouse model of graft-versus-human disease (GVHD), which is superior to the use of TNF inhibitor etanercept (Zhang B. et al., 2018). MSC-Exo suppresses the production of TNF-α and IL-1β and increases the production of TGF-β, IL-10, IL-33, and IL-34, which promotes a T-cell profile with an enhanced Th-2 response, reduced Th-17, and amplified Treg cells (Chen et al., 2016; Zhu D. et al., 2022). In addition, human ADMSC-Exo and umbilical cord blood-derived MSCs-derived microvesicles are rich in tolerogenic surface molecules, which inhibit autoreactive lymphocyte proliferation and induce CD4^+^CD25^+^FOXP3^+^ Treg cells in both *in vitro* studies and animal models (Blazquez et al., 2014; Kilpinen et al., 2013; Mokarizadeh et al., 2012; Nojehdehi et al., 2018). These immunomodulatory effects become even stronger when MSCs are pre-stimulated with inflammatory cues (Kilpinen et al., 2013; Mokarizadeh et al., 2012). MSC-EVs do not completely replicate the activity of their parent MSCs. In contrast to MSCs, which mainly suppress the proliferation of T-cells, MSC-EVs may induce apoptosis in effector T-cells while increasing Treg cell percentage, an immunologic signature unique to EVs (Del Fattore et al., 2015). As discussed earlier, miR-loaded MSC-EVs play a crucial role in immune response. miR-181a-enriched MSC-Exo weakened immune and inflammatory responses, suppressed some genes like c-Fos, promoted Treg cell formation, and prevented myocardial damage in MIRI models (Wei et al., 2019). However, the role of miR-181a is not uniformly protective. For instance, elevated miR-181a in other exosome sources is linked to increased inflammation, hypertrophy, and fibrosis after MI, and its overexpression in MSCs abolishes their anti-inflammatory effects *in vivo* (Vaskova et al., 2020; Liu et al., 2012). These contradictory findings highlight the context-dependent role of miR-181a, where its therapeutic benefit appears to depend on the cellular origin of EVs, the pathological stage of myocardial injury, and the level of miR expression.

MSC-EVs also influence humoral immunity. Budoni et al., (2013) showed that MSC-EVs can replicate MSC-mediated suppression of B cells and may serve as a safer, standardized alternative to cell therapy (Budoni et al., 2013). *In vitro* studies have shown that MSC-Exo inhibit the proliferation of activated peripheral blood mononuclear cells (PBMCs), T cells, and B cells, and in B lymphocytes they alter the expression of more than 180 genes involved in migration, differentiation, immune function, and hemostasis, which are reflected by reduced IgM production (Khare et al., 2018). However, other studies indicate that MSC-driven modulation of naïve B cells and IL-10-producing Breg cells depends more on soluble factors than on EVs alone, pointing to a combined or context-dependent mechanism (Carreras-Planella et al., 2019). Although MSC-EVs can replicate some MSC-mediated suppression of B-cell activity, the relative contributions of EVs versus soluble factors in regulating naïve and Breg cells remain unclear. Other work shows that BMMSC-Exo enriched in miR-214 protect CSCs from oxidative stress-induced apoptosis and limit ROS accumulation (Wang Y. et al., 2018). Moreover, combination therapies may augment these advantages: for instance, combining cold water therapy with ADMSC-Exo provided greater protection against MIRI than either treatment alone (Chai et al., 2019).

Comparisons between MSCs and their EVs reveal some functional differences. Conforti et al. (2014) confirmed that MSCs were more effective in comparison to their microvesicles in T-cell proliferation suppression and B-cell differentiation prevention (Conforti et al., 2014). These differences likely result from challenges in standardizing microvesicles preparations or matching equivalent MSC and microvesicles doses. In comparison, another study shows that MSC-Exo outperformed whole MSCs by reducing fibrosis and inflammation and improving cardiac function. These superior effects were attributed largely to their specialized miR cargo (Shao et al., 2017). Although both EVs and MSCs show cardioprotective potential, their relative efficacy is still unclear. Overall, MSC-EVs work as immune stabilizers for MI, but their process variation and random potency remain a major concern (de Abreu et al., 2020; Forsberg et al., 2020; Hassanzadeh et al., 2021).

### 4.2 EVs from cardiac-lineage cells

**FIGURE 4:**
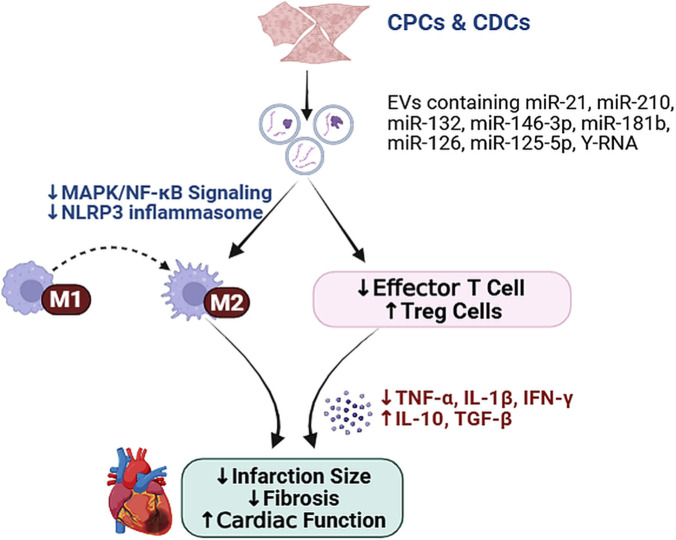
Immunomodulatory effects of EVs derived from cardiac progenitor cells (CPCs) and cardiosphere-derived cells (CDCs) after MI.

Administration of human embryonic stem cell-derived cardiovascular progenitor cells (hESC-CPCs) during the early stages of myocardial injury has been shown to promote a more reparative immune environment within the heart. These progenitor cells drive macrophages towards a healing phenotype largely through the secretion of IL-4 and IL-13, as demonstrated in C57BL/6 and BALB/c wild-type mouse models of MI (Wang et al., 2019). Over the past decade, a growing body of work has highlighted the importance of EV-based communication as a primary driver of progenitor cell-mediated cardiac recovery (Lang et al., 2016; Maring et al., 2019). Loyer et al., (2018) proved that after acute infarction, the heart itself increases the production of EVs, both exosomes and microvesicles, which accumulate in the ischemic zone and are rapidly taken up by infiltrating monocytes and subsequently shape the inflammatory milieu of the injured myocardium (Loyer et al., 2018), as shown in Figure 4 **.** In porcine models of both acute and chronic MI, direct intramyocardial injection of CDC-derived exosomes (CDC-Exo) has consistently reduced scar formation, limited pathological ventricular remodeling, and improved left ventricular ejection fraction (LVEF) (Gallet et al., 2017). MSCs, CPCs and CDCs produce EVs that are enriched with a variety of regulatory miRs, including miR-21, miR-210, miR-132, miR-146a-3p, miR-208a/b, and miR-499 (Adachi et al., 2010; Barile et al., 2014; Maleki et al., 2022; Boldin et al., 2011; Zheng et al., 2020). These miRs collectively influence cardiomyocyte survival, migration, and proliferation, and they help restrain fibrosis, inflammation, and harmful immune activation. Notably, their expression patterns shift considerably when CSCs are exposed to exosomes secreted by MSCs (Barile et al., 2014; Zhang et al., 2016). Exosomes from CDCs containing miR-146a, miR-181b, and miR-126 reduce the CD68^+^ macrophages and enhance the anti-inflammatory effect in both Wistar-Kyoto rats and Yucatan mini-pig models of MIRI (de Couto et al., 2017). In addition, specific CDC-EV cargo, particularly fragments of Y RNA, can trigger IL-10-producing macrophages, which protect cardiomyocytes from oxidative stress *in vitro* and lessen infarct size *in vivo* (Cambier et al., 2017). A comparative study demonstrated that CPC-Exo and cardiac stromal cell-derived EVs offer stronger cardioprotection than those from bone marrow cells by reducing apoptosis, limiting scar size, and improving heart function (Barile et al., 2018; Czosseck et al., 2024). Compared with MSC-EVs, CDC-EVs show less systemic immunosuppression but stronger cardiac specificity, which may reduce off-target immune effects. However, their clinical scalability is limited by the availability, lifespan, and expansion capacity of CPCs (van Berlo et al., 2014).

### 4.3 EVs from induced pluripotent stem cell derivatives

Available primary stem cells have been a limiting factor in efforts to translate EV-based therapies into clinical use. These cells are difficult to grow in large numbers, grow only moderately in culture, and tend to drift into senescence with prolonged passaging. MSCs produced by iPSCs (iMSCs) can provide a viable solution to these production problems. Because iMSCs can be generated on a large scale and maintain robust proliferative capacity, they provide a reliable and abundant source of EVs with strong immunomodulatory potential, and their functional profile is similar to human UCMSCs (Buitrago et al., 2024). Recent studies demonstrated that iMSC-derived EVs (iMSC-EVs) promote the transfer of macrophage M1 inflammatory to an M2 anti-inflammatory phenotype via regulation of p38 MAPK signaling (Ye et al., 2023). Similarly, Peng et al. (2023) proved that iMSC-EVs affect macrophages by producing miR-125b-5p that inhibit downstream NF-kB signaling (Peng et al., 2023; Wang et al., 2014). These mechanisms assist iMSCs and their EVs in T-cell growth suppression, and improve tissue repair (Buitrago et al., 2024).

The immunosuppressive effect of iMSC-EVs can be further improved via preconditioning to an inflammatory condition (Buitrago et al., 2024). These findings make iMSC-EVs a vital tool for MSC therapies. However, variation among different batches creates a negative impact on iMSC-EVs (Tertel et al., 2023). In addition to having an endogenous immunomodulatory effect, EVs produced by iMSCs can also serve as targeted delivery vehicles (Ju et al., 2017). *In vivo* findings also point out the therapeutic potential of iMSC-EVs. EV therapy in a mouse MI model improved the left ventricle’s function, increased perfusion, maintained viable myocardium, and decreased hypertrophy more than direct transplantation of iMSCs without causing teratoma (Adamiak et al., 2018). These advantages can be enhanced with the help of delivery technologies, for example, hydrogel patches with slow release of EVs from iPSC-derived cardiomyocytes significantly enhanced cardiac repair after MI in Sprague-Dawley rats (Liu et al., 2018). In other studies, it is also reported that exosomes released by iPSC-differentiated cells also regulate autophagy and promote myocardial regeneration (Santoso et al., 2020; Tachibana et al., 2017). iMSC-EVs represent a translationally scalable immune-modulatory platform, but their long-term safety, stability, and cardiac specificity require further validation.

Although the therapeutic actions of stem cell-EVs on macrophages, monocytes, T cells, and DCs are also fully documented, the magnitude to which these interventions influence the other population of immune cells has not been well defined yet and highlights the need for additional research. Table 2 summarizes the main EV sources, their miR cargo, molecular targets, and immunomodulatory effects in MI.

**TABLE 2:** Comparative immunomodulatory effects of stem cell-derived extracellular vesicles (EVs) in myocardial infarction (MI).

EV source	Representative cargo	Major immune targets	Evidence level	Key therapeutic advantages	Current limitations	Ref.
Mesenchymal Stem Cell-derived EVs (MSC-EVs)	miR-21, miR-146a, miR-182, miR-125a/b, miR-181a, miR-223, prostaglandin E2, TGF-β, IL-10	Macrophages, dendritic cells, T cells, neutrophils	Extensive in vitro and multiple preclinical MI studies	Extensively studied; broad therapeutic applicability, potent immunomodulation; promotes M2 macrophage polarization; reduces inflammatory cytokines; relatively low immunogenicity	Donor-dependent cargo variability; heterogeneity in isolation methods; limited large-scale standardization	Kou et al. (2022)
Cardiosphere-derived Cell EVs (CDC-EVs)/Cardiac Lineage Cell-derived EVs	miR-21, miR-126, miR-210, miR-132, miR-146a, miR-181b, Y-RNA fragments	Macrophages, inflammatory monocytes, fibroblasts, inflammatory cardiac microenvironment	Primarily preclinical animal studies in myocardial infarction models	Cardiac tissue specificity; anti-fibrotic effects; promotes cardiomyocyte survival and repair; Strong anti-inflammatory effects	Limited cell availability; lower scalability compared with MSCs; donor and tissue-source variability, complex manufacturing	Gallet et al. (2017)
Induced Pluripotent Stem Cell-derived EVs (iPSC-EVs/iMSC-EVs)	miR-24, miR-125b-5p, regenerative miRs, growth factors	Macrophages, T cells, reparative signaling pathways	Emerging preclinical evidence	High regenerative potential; scalable cell source; personalized medicine potential, customizable engineering potential	Risk of residual pluripotency-related factors, safety and quality-control concerns; limited long-term translational data	Xiao et al. (2015)

## 5 Recent innovations in stem cell-derived EV therapy

Interest in EVs continues to grow as the global EV market expands, driven by rapid development in EV-based diagnostics, regenerative treatments, and engineered vesicle drug-delivery systems (Cheng and Kalluri, 2023). Efforts to improve MSC-EV-based therapies have explored combinatorial strategies, preconditioning approaches, and genetic engineering. Inflammatory licensing of MSCs appears to enhance their immunoregulatory output (Ti et al., 2015). Di Trapani et al., (2016) reported that EVs released from preconditioned MSCs can activate resting MSCs to adopt a strongly immunosuppressive phenotype, even though the EVs themselves do not directly inhibit T-cell division (Di Trapani et al., 2016). Further *in vitro* studies demonstrate that exosomes from TGF-β-, TNF-α- or IFN-γ-preconditioned iMSCs exhibit heightened immunosuppressive activity, including elevated IDO (indoleamine 2,3-dioxygenase) levels and the ability to induce Treg differentiation, even in the presence of activated macrophages (Cassano et al., 2018; Zhang Q. et al., 2018; Ramos et al., 2022). In a similar manner, IL-1β-licensed human UCMSCs and BMMSCs secrete exosomes enriched in miR-146a that effectively promote M2 macrophage polarization via downregulation of NF-κB signaling and reduce post-MI inflammation and cardiomyocyte apoptosis (Xu et al., 2019; Song et al., 2017). Moreover, multiple studies have shown that exosomes from hypoxia-treated MSCs provide stronger anti-inflammatory and cardioprotective effects *in vivo* models of MIRI (Liu et al., 2019; Lo Sicco et al., 2017; Zhu J. et al., 2018). Similarly, recent *in vitro* study by Zhang et al. (2026) demonstrated that after hypoxia/reoxygenation injury, heart cells release EVs carrying high levels of miR-199b-3p. These vesicles are taken up by macrophages, where the miR suppresses Fgl2 (fibrinogen-like protein 2), dampens inflammatory signaling, reduces pro-inflammatory activation, and improves heart function in mice with MI (Zhang et al., 2026). In addition, recent studies have shown that preconditioning MSC-Exo with natural compounds or herbal bioactive agents can also enhance their immunomodulatory effects in myocardial repair (Li et al., 2023; Sha et al., 2024).

**FIGURE 5:**
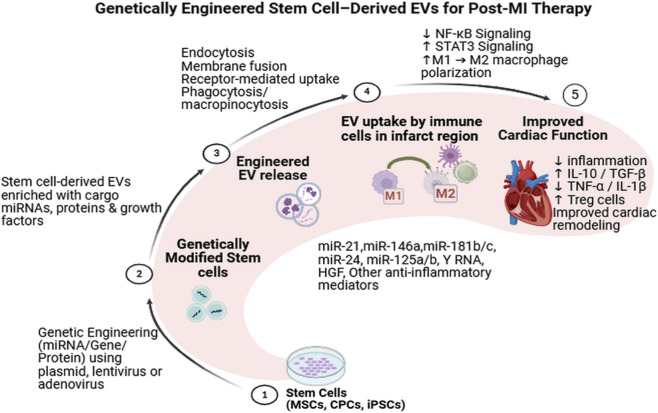
Mechanism of genetically engineered stem cell-derived EVs for MI therapy.

In the meantime, bioengineering now allows the alteration of EV surfaces or loading vesicles with therapeutic molecules to enhance biodistribution and potency (Feng et al., 2021; Ma et al., 2021). As an example, macrophage and T cell recruitment were significantly inhibited by platelet-derived EVs loaded with the NLRP3 inhibitor (MCC950) in injured tissue (Ma et al., 2021). Similarly, the exosomes containing the ICAM-1 siRNA (small interfering RNA) were able to successfully enter endothelial cells, silence the expression of ICAM-1, and lower the neutrophil adhesion and inflammation, proving the great potential of the future approach towards treating vascular inflammation (Ju et al., 2017). Moreover, biomaterials such as hydrogels and microspheres further enhance their stability and allow controlled, long-term release in the body. These engineered systems have shown strong cardioprotective effects, including reduced cell death, decreased fibrosis, improved blood vessel formation, and better heart function recovery (Li et al., 2026). Engineered stem cell-EVs carrying specific miRs can regulate disease-associated signaling pathways, enhance cellular repair mechanisms, and reduce tissue damage in experimental models. These findings further support the growing interest in EVs as versatile and targeted drug-delivery platforms. Similarly, exosomes from genetically modified stem cells can improve heart repair after MI, as shown in Figure 5 **.**

In addition to natural exosomes, synthetic or biomimetic vesicles that can specifically mimic exosome signaling pathways have been demonstrated to be useful therapeutically. As an example, synthetic neutrophil apoptotic bodies (eNABs), which mimic the characteristics of naturally apoptotic neutrophils, selectively targeted macrophages and reprogrammed them towards anti-inflammatory activity, reducing TNF-α and IL-6 levels and increasing TGF-β and IL-10, thereby enhancing myocardial repair after MI (Bao et al., 2022). Similarly, engineered chimeric apoptotic bodies derived from stem cells provide a flexible system in which regenerative and immunoregulatory capability can be altered by loading and surface modifications of these apoptotic bodies (Dou et al., 2020).

There are other emerging technologies that demonstrate the rate at which EV-based therapies are developing. Among them is the creation of stents releasing MSC-Exo that enhance the polarization of macrophages towards a reparative state, reduce inflammation, and improve vascular healing after ischemic injury in rat models (Hu et al., 2021). Another method is Stem Cell-Exo Nebulization Therapy (SCENT), which delivers stem cell-EVs directly to the injured myocardium via non-invasive inhalation. The use of this strategy has been demonstrated to improve cardiac function, decrease fibrosis, and promote cardiomyocyte proliferation after MI (Li et al., 2024). One of the most powerful recent new technologies is the wireless bioelectronic ePOWER patch. This instrument produces EVs at the site of infarction by electrically stimulating macrophages. The system generated up to 24-fold more functional EVs per cell than the traditional culture method and caused a large reduction in myocardial injury and improved ventricular performance (Fu et al., 2025). This cell-free and localized *in situ* EV generation platform provides a programmable, local, and therapeutic treatment of MI and possibly other disorders, potentially evading the systemic delivery approach and increasing the availability of therapeutic EVs (Fu et al., 2025). Additionally, electrospinning and 3D bioprinting can be used as biofabrication techniques to scale the production of EV-producing cardiac patches that can be clinically translated into use (Fu et al., 2025). Similar studies show that 3D-derived exosomes have enhanced therapeutic potential, contributing to improved disease-related pathway regulation, reduced pathological burden, and better functional recovery in experimental models (Pan et al., 2025).

## 6 Challenges in EV-Based clinical applications

The clinical translation of EV-based therapies remains hindered by several obstacles, despite accelerated development. One of the key impediments to exosome-based cardiac therapy is the challenge of generating enough EVs for clinical-scale therapy. Mechanical extrusion has also been suggested as a possible solution, where nanovesicles closely related to natural MSC-EVs can be obtained, potentially up to 20 times higher quantities (Wang et al., 2021). Nonetheless, EV formation is much more conditional on the physiological condition and culture environment of parent cells (Menard et al., 2013). Consequently, the molecular cargo and biological activity of EV preparations also appear highly variable (de Abreu et al., 2020; Forsberg et al., 2020; Hassanzadeh et al., 2021). This has been proved by studies that various subsets of MSC-EV batches frequently exhibit different immunomodulatory functions, and it is not possible to predict the potency of a MSC-EV’s batch with a reliable marker (Bauer et al., 2023; Kordelas et al., 2019; Madel et al., 2023). The lack of standardized methods for isolating and characterizing EVs makes results difficult to reproduce and compare across studies (Lener et al., 2015; Théry et al., 2018). In addition, the complex immune environment after MI makes EV effects variable and sometimes unpredictable (Boulanger et al., 2017; Davidson et al., 2018). There are also safety concerns, as EVs may cause unintended immune activation or suppression (Kamerkar et al., 2017; Tkach and Théry, 2016). Moreover, limited understanding of how EVs regulate the immune system makes it harder to develop reliable and targeted therapies (Boulanger et al., 2017; Toh et al., 2017).

Other complications are contamination risks, such as *mycoplasma*, viral particles, endotoxins, or medium-derived impurities, which require rigorous quality control to ensure the safety of these EVs (de Abreu et al., 2020; Takakura et al., 2024; Dou et al., 2020). Moreover, once EVs reach their target cells, they are engulfed by lysosomes, and their therapeutic cargoes are destroyed prior to delivering any functional impact (Zheng et al., 2019). Certain attempts to chemically modify or load EVs to enhance targeting are also associated with flaws, for example, electroporation may lead to RNA aggregation, loading inefficiency, and change in vesicle integrity (Kooijmans et al., 2013; Tian et al., 2018). Lastly, EVs have been highly implicated in normal and pathological signaling mechanisms. EVs produced by tumors are also known to drive cancer proliferation and metastasis, and careful purification and safety measures should be undertaken to ensure that improper risks are avoided (Takakura et al., 2024; Kosaka et al., 2016). These challenges complicate the interpretation of results across studies and underscore the need for systematic strategies to enhance EV therapeutics.

## 7 Future directions and conclusion

Stem cell-EVs suppress unwanted inflammation, reprogram macrophages to reduce inflammation, promote the growth of Treg cells, and facilitate cardiomyocyte regeneration. Their pleiotropic activities, including miRs, cytokines, lipids, and signaling molecules, even surpass those of parent stem cells, and overcome the shortcomings of cell transplantation. New developments in EV bioengineering, preconditioning, and targeted delivery also help to enhance their therapeutic capabilities, and provide approaches to achieve greater stability, tissue specificity, and homeostatic ways of immunomodulation. However, several challenges, particularly the heterogeneity of EV cargo, continue to impede their clinical implementation. The goal of future research should involve establishing regular and standardized approaches to manufacture and characterize stem cell-EVs in post-MI repair. Moreover, researchers also need the development of reliable potency assays or biomarker-based readouts, which would enable the determination of the effectiveness/potency of EVs in regulating immune reactions and preventing fibrosis in the post-MI period.

## References

[B1] Adachi T. Nakanishi M. Otsuka Y. Nishimura K. Hirokawa G. Goto Y. (2010). Plasma microRNA 499 as a biomarker of acute myocardial infarction. Clin. Chem. 56 (7), 1183–1185. 10.1373/clinchem.2010.144121 20395621

[B2] Adamiak M. Cheng G. Bobis-Wozowicz S. Zhao L. Kedracka-Krok S. Samanta A. (2018). Induced pluripotent stem cell (iPSC)-Derived extracellular vesicles are safer and more effective for cardiac repair than iPSCs. Circulation Res. 122 (2), 296–309. 10.1161/CIRCRESAHA.117.311769 29118058 PMC5775034

[B3] Aggarwal S. Pittenger M. F. (2005). Human mesenchymal stem cells modulate allogeneic immune cell responses. Blood 105 (4), 1815–1822. 10.1182/blood-2004-04-1559 15494428

[B4] Alvarez-Erviti L. Seow Y. Yin H. Betts C. Lakhal S. Wood M. J. A. (2011). Delivery of siRNA to the mouse brain by systemic injection of targeted exosomes. Nat. Biotechnol. 29 (4), 341–345. 10.1038/nbt.1807 21423189

[B5] Anzai A. Anzai T. Nagai S. Maekawa Y. Naito K. Kaneko H. (2012). Regulatory role of dendritic cells in postinfarction healing and left ventricular remodeling. Circulation 125 (10), 1234–1245. 10.1161/circulationaha.111.052126 22308302

[B6] Arslan F. Lai R. C. Smeets M. B. Akeroyd L. Choo A. Aguor E. N. E. (2013). Mesenchymal stem cell-derived exosomes increase ATP levels, decrease oxidative stress and activate PI3K/Akt pathway to enhance myocardial viability and prevent adverse remodeling after myocardial ischemia/reperfusion injury. Stem Cell Res. 10 (3), 301–312. 10.1016/j.scr.2013.01.002 23399448

[B7] Azevedo R. I. Minskaia E. Fernandes-Platzgummer A. Vieira A. I. S. da Silva C. L. Cabral J. M. S. (2020). Mesenchymal stromal cells induce regulatory T cells via epigenetic conversion of human conventional CD4 T cells *in vitro* . Stem Cells Dayt. Ohio 38 (8), 1007–1019. 10.1002/stem.3185 32352186 PMC7497276

[B8] Bajpai G. Bredemeyer A. Li W. Zaitsev K. Koenig A. L. Lokshina I. (2019). Tissue resident CCR2− and CCR2+ cardiac macrophages differentially orchestrate monocyte recruitment and fate specification following myocardial injury. Circulation Research 124 (2), 263–278. 10.1161/CIRCRESAHA.118.314028 30582448 PMC6626616

[B9] Bao L. Dou G. Tian R. Lv Y. Ding F. Liu S. (2022). Engineered neutrophil apoptotic bodies ameliorate myocardial infarction by promoting macrophage efferocytosis and inflammation resolution. Bioact. Mater. 9, 183–197. 10.1016/j.bioactmat.2021.08.008 34820565 PMC8586716

[B10] Barile L. Lionetti V. Cervio E. Matteucci M. Gherghiceanu M. Popescu L. M. (2014). Extracellular vesicles from human cardiac progenitor cells inhibit cardiomyocyte apoptosis and improve cardiac function after myocardial infarction. Cardiovasc. Res. 103 (4), 530–541. 10.1093/cvr/cvu167 25016614

[B11] Barile L. Cervio E. Lionetti V. Milano G. Ciullo A. Biemmi V. (2018). Cardioprotection by cardiac progenitor cell-secreted exosomes: role of pregnancy-associated plasma protein-A. Cardiovasc. Res. 114 (7), 992–1005. 10.1093/cvr/cvy055 29518183

[B12] Bartholomew A. Sturgeon C. Siatskas M. Ferrer K. McIntosh K. Patil S. (2002). Mesenchymal stem cells suppress lymphocyte proliferation *in vitro* and prolong skin graft survival *in vivo* . Exp. Hematol. 30 (1), 42–48. 10.1016/s0301-472x(01)00769-x 11823036

[B13] Bauer F. N. Tertel T. Stambouli O. Wang C. Dittrich R. Staubach S. (2023). CD73 activity of mesenchymal stromal cell-derived extracellular vesicle preparations is detergent-resistant and does not correlate with immunomodulatory capabilities. Cytotherapy 25 (2), 138–147. 10.1016/j.jcyt.2022.09.006 36244910

[B14] Ben-Mordechai T. Holbova R. Landa-Rouben N. Harel-Adar T. Feinberg M. S. Abd Elrahman I. (2013). Macrophage subpopulations are essential for infarct repair with and without stem cell therapy. J. Am. Coll. Cardiol. 62 (20), 1890–1901. 10.1016/j.jacc.2013.07.057 23973704

[B15] Bian S. Zhang L. Duan L. Wang X. Min Y. Yu H. (2014). Extracellular vesicles derived from human bone marrow mesenchymal stem cells promote angiogenesis in a rat myocardial infarction model. J. Mol. Med. Berlin, Ger. 92 (4), 387–397. 10.1007/s00109-013-1110-5 24337504

[B16] Blackwell J. A. Stanford K. I. (2023). Exercise-induced intertissue communication: adipose tissue and the heart. Curr. Opin. Physiology 31, 100626. 10.1016/j.cophys.2022.100626 PMC9802643 36588657

[B17] Blanco-Domínguez R. de la Fuente H. Rodríguez C. Martín-Aguado L. Sánchez-Díaz R. Jiménez-Alejandre R. (2022). CD69 expression on regulatory T cells protects from immune damage after myocardial infarction. J. Clin. Investigation 132 (21), e152418. 10.1172/jci152418 PMC9621142 36066993

[B18] Blazquez R. Sanchez-Margallo F. M. de la Rosa O. Dalemans W. Alvarez V. Tarazona R. (2014). Immunomodulatory potential of human adipose mesenchymal stem cells derived exosomes on *in vitro* stimulated T cells. Front. Immunol. 5. 556. 10.3389/fimmu.2014.00556 25414703 PMC4220146

[B19] Boldin M. P. Taganov K. D. Rao D. S. Yang L. Zhao J. L. Kalwani M. (2011). miR-146a is a significant brake on autoimmunity, myeloproliferation, and cancer in mice. J. Experimental Medicine 208 (6), 1189–1201. 10.1084/jem.20101823 21555486 PMC3173243

[B20] Bönner F. Borg N. Jacoby C. Temme S. Ding Z. Flögel U. (2013). Ecto-5’-nucleotidase on immune cells protects from adverse cardiac remodeling. Circulation Res 113 (3), 301–312. 10.1161/CIRCRESAHA.113.300180 23720442

[B21] Borg N. Alter C. Görldt N. Jacoby C. Ding Z. Steckel B. (2017). CD73 on T cells orchestrates cardiac wound healing after myocardial infarction by purinergic metabolic reprogramming. Circulation 136 (3), 297–313. 10.1161/circulationaha.116.023365 28432149

[B22] Borges F. T. Melo S. A. Özdemir B. C. Kato N. Revuelta I. Miller C. A. (2013). TGF-β1–Containing exosomes from injured epithelial cells activate fibroblasts to initiate tissue regenerative responses and fibrosis. J. Am. Soc. Nephrol. JASN 24 (3), 385–392. 10.1681/ASN.2012101031 23274427 PMC3582210

[B23] Boulanger C. M. Loyer X. Rautou P. E. Amabile N. (2017). Extracellular vesicles in coronary artery disease. Nat. Rev. Cardiol. 14 (5), 259–272. 10.1038/nrcardio.2017.7 28150804

[B24] Bruno S. Grange C. Deregibus M. C. Calogero R. A. Saviozzi S. Collino F. (2009). Mesenchymal stem cell-derived microvesicles protect against acute tubular injury. J. Am. Soc. Nephrol. JASN 20 (5), 1053–1067. 10.1681/asn.2008070798 19389847 PMC2676194

[B25] Bruno S. Grange C. Collino F. Deregibus M. C. Cantaluppi V. Biancone L. (2012). Microvesicles derived from mesenchymal stem cells enhance survival in a lethal model of acute kidney injury. PLoS ONE 7 (3), e33115. 10.1371/journal.pone.0033115 22431999 PMC3303802

[B26] Budoni M. Fierabracci A. Luciano R. Petrini S. Di Ciommo V. Muraca M. (2013). The immunosuppressive effect of mesenchymal stromal cells on B lymphocytes is mediated by membrane vesicles. Cell Transplant. 22 (2), 369–379. 10.3727/096368911X582769 23433427

[B27] Buitrago J. C. Morris S. L. Backhaus A. Kaltenecker G. Kaipa J. M. Girard C. (2024). Unveiling the immunomodulatory and regenerative potential of iPSC-derived mesenchymal stromal cells and their extracellular vesicles. Sci. Rep. 14 (1), 24098. 10.1038/s41598-024-75956-3 39407038 PMC11480492

[B28] Cambier L. de Couto G. Ibrahim A. Echavez A. K. Valle J. Liu W. (2017). Y RNA fragment in extracellular vesicles confers cardioprotection via modulation of IL‐10 expression and secretion. EMBO Mol. Med. 9 (3), 337–352. 10.15252/emmm.201606924 28167565 PMC5331234

[B29] Caplan A. I. Dennis J. E. (2006). Mesenchymal stem cells as trophic mediators. J. Cell. Biochem. 98 (5), 1076–1084. 10.1002/jcb.20886 16619257

[B30] Carreras-Planella L. Monguió-Tortajada M. Borràs F. E. Franquesa M. (2019). Immunomodulatory effect of MSC on B cells is independent of secreted extracellular vesicles. Front. Immunol. 10, 1288. 10.3389/fimmu.2019.01288 31244839 PMC6563675

[B31] Cassano J. M. Schnabel L. V. Goodale M. B. Fortier L. A. (2018). Inflammatory licensed equine MSCs are chondroprotective and exhibit enhanced immunomodulation in an inflammatory environment. Stem Cell Res. and Ther. 9, 82. 10.1186/s13287-018-0840-2 29615127 PMC5883371

[B32] Chai H.-T. Sheu J. J. Chiang J. Y. Shao P. L. Wu S. C. Chen Y. L. (2019). Early administration of cold water and adipose derived mesenchymal stem cell derived exosome effectively protects the heart from ischemia-reperfusion injury. Am. J. Transl. Res. 11 (9), 5375–5389. 31632517 PMC6789220

[B33] Chen T. S. Lai R. C. Lee M. M. Choo A. B. H. Lee C. N. Lim S. K. (2010). Mesenchymal stem cell secretes microparticles enriched in pre-microRNAs. Nucleic Acids Res. 38 (1), 215–224. 10.1093/nar/gkp857 19850715 PMC2800221

[B34] Chen W. Huang Y. Han J. Yu L. Li Y. Lu Z. (2016). Immunomodulatory effects of mesenchymal stromal cells-derived exosome. Immunol. Res. 64 (4), 831–840. 10.1007/s12026-016-8798-6 27115513

[B35] Chen Y. Zhao Y. Chen W. Xie L. Zhao Z. A. Yang J. (2017). MicroRNA-133 overexpression promotes the therapeutic efficacy of mesenchymal stem cells on acute myocardial infarction. Stem Cell Res. and Ther. 8, 268. 10.1186/s13287-017-0722-z 29178928 PMC5702098

[B36] Chen Q. Liu Y. Ding X. Li Q. Qiu F. Wang M. (2020). Bone marrow mesenchymal stem cell-secreted exosomes carrying microRNA-125b protect against myocardial ischemia reperfusion injury via targeting SIRT7. Mol. Cell. Biochem. 465 (1), 103–114. 10.1007/s11010-019-03671-z 31858380 PMC6955239

[B37] Chen X. Yang F. (2024a). “Classification and nomenclature of extracellular vesicles,” in Extracellular Vesicles: From Bench to Bedside. Editors Wang, Q. Zheng L. (Singapore: Springer Nature), 3–7.

[B38] Chen R. Zhang H. Tang B. Luo Y. Yang Y. Zhong X. (2024b). Macrophages in cardiovascular diseases: molecular mechanisms and therapeutic targets. Signal Transduct. Target. Ther. 9 (1), 130. 10.1038/s41392-024-01840-1 38816371 PMC11139930

[B39] Cheng K. Kalluri R. (2023). Guidelines for clinical translation and commercialization of extracellular vesicles and exosomes based therapeutics. Extracell. Vesicle 2, 100029. 10.1016/j.vesic.2023.100029

[B40] Chiossone L. Conte R. Spaggiari G. M. Serra M. Romei C. Bellora F. (2016). Mesenchymal stromal cells induce peculiar alternatively activated macrophages capable of dampening both innate and adaptive immune responses. Stem Cells Dayt. Ohio 34 (7), 1909–1921. 10.1002/stem.2369 27015881

[B41] Choo E. H. Lee J. H. Park E. H. Park H. E. Jung N. C. Kim T. H. (2017). Infarcted myocardium-primed dendritic cells improve remodeling and cardiac function after myocardial infarction by modulating the regulatory T cell and macrophage polarization. Circulation 135 (15), 1444–1457. 10.1161/CIRCULATIONAHA.116.023106 28174192

[B42] Chugh A. R. Beache G. M. Loughran J. H. Mewton N. Elmore J. B. Kajstura J. (2012). Administration of cardiac stem cells in patients with ischemic cardiomyopathy (the SCIPIO trial): surgical aspects and interim analysis of myocardial function and viability by magnetic resonance. Circulation 126 (1), S54–S64. 10.1161/circulationaha.112.092627 22965994 PMC3448934

[B43] Collino F. Deregibus M. C. Bruno S. Sterpone L. Aghemo G. Viltono L. (2010). Microvesicles derived from adult human bone marrow and tissue specific mesenchymal stem cells shuttle selected pattern of miRNAs. PLOS ONE 5 (7), e11803. 10.1371/journal.pone.0011803 20668554 PMC2910725

[B44] Colombo M. Raposo G. Théry C. (2014). Biogenesis, Secretion, and Intercellular Interactions of Exosomes and Other Extracellular Vesicles. Annu Rev Cell Dev Biol. 30:255-289. 10.1146/annurev-cellbio-101512-122326 25288114

[B45] Conforti A. Scarsella M. Starc N. Giorda E. Biagini S. Proia A. (2014). Microvescicles derived from mesenchymal stromal cells are not as effective as their cellular counterpart in the ability to modulate immune responses *in vitro* . Stem Cells Dev. 23 (21), 2591–2599. 10.1089/scd.2014.0091 24937591 PMC4201301

[B46] Corcione A. Benvenuto F. Ferretti E. Giunti D. Cappiello V. Cazzanti F. (2006). Human mesenchymal stem cells modulate B-cell functions. Blood 107 (1), 367–372. 10.1182/blood-2005-07-2657 16141348

[B47] Curato C. Slavic S. Dong J. Skorska A. Altarche-Xifró W. Miteva K. (2010). Identification of noncytotoxic and IL-10-producing CD8+AT2R+ T cell population in response to ischemic heart injury. J. Immunol. Baltim. Md. 1950 185 (10), 6286–6293. 10.4049/jimmunol.0903681 20935205

[B48] Czosseck A. Chen M. M. Hsu C. C. Shamrin G. Meeson A. Oldershaw R. (2024). Extracellular vesicles from human cardiac stromal cells up-regulate cardiomyocyte protective responses to hypoxia. Stem Cell Res. and Ther. 15 (1), 363. 10.1186/s13287-024-03983-y 39396003 PMC11470622

[B49] Davidson S. M. Takov K. Yellon D. M. (2017). Exosomes and cardiovascular protection. Cardiovasc. Drugs Ther. 31 (1), 77–86. 10.1007/s10557-016-6698-6 27796607 PMC5346599

[B50] Davidson S. M. Riquelme J. A. Zheng Y. Vicencio J. M. Lavandero S. Yellon D. M. (2018). Endothelial cells release cardioprotective exosomes that may contribute to ischaemic preconditioning. Sci. Rep. 8, 15885. 10.1038/s41598-018-34357-z 30367147 PMC6203728

[B51] de Abreu R. C. Fernandes H. da Costa Martins P. A. Sahoo S. Emanueli C. Ferreira L. (2020). Native and bioengineered extracellular vesicles for cardiovascular therapeutics. Nat. Rev. Cardiol. 17 (11), 685–697. 10.1038/s41569-020-0389-5 32483304 PMC7874903

[B52] de Couto G. Gallet R. Cambier L. Jaghatspanyan E. Makkar N. Dawkins J. F. (2017). Exosomal microRNA transfer into macrophages mediates cellular postconditioning de Couto: exosomal RNA transfer modulates macrophages. Circulation 136 (2), 200–214. 10.1161/circulationaha.116.024590 28411247 PMC5505791

[B53] DeBerge M. Yu S. Dehn S. Ifergan I. Yeap X. Y. Filipp M. (2020). Monocytes prime autoreactive T cells after myocardial infarction. Am. J. Physiology - Heart Circulatory Physiology 318 (1), H116–H123. 10.1152/ajpheart.00595.2019 31809213 PMC6985803

[B54] Del Fattore A. Luciano R. Pascucci L. Goffredo B. M. Giorda E. Scapaticci M. (2015). Immunoregulatory effects of mesenchymal stem cell-derived extracellular vesicles on T lymphocytes. Cell Transplant. 24 (12), 2615–2627. 10.3727/096368915X687543 25695896

[B55] Deng S. Zhou X. Ge Z. Song Y. Wang H. Liu X. (2019). Exosomes from adipose-derived mesenchymal stem cells ameliorate cardiac damage after myocardial infarction by activating S1P/SK1/S1PR1 signaling and promoting macrophage M2 polarization. Int. J. Biochem. and Cell Biol. 114, 105564. 10.1016/j.biocel.2019.105564 31276786

[B56] Di Trapani M. Bassi G. Midolo M. Gatti A. Kamga P. T. Cassaro A. (2016). Differential and transferable modulatory effects of mesenchymal stromal cell-derived extracellular vesicles on T, B and NK cell functions. Sci. Rep. 6 (1), 24120. 10.1038/srep24120 27071676 PMC4829861

[B57] Dick S. A. Macklin J. A. Nejat S. Momen A. Clemente-Casares X. Althagafi M. G. (2019). Self-renewing resident cardiac macrophages limit adverse remodeling following myocardial infarction. Nat. Immunol. 20 (1), 29–39. 10.1038/s41590-018-0272-2 30538339 PMC6565365

[B58] Djouad F. Charbonnier L. M. Bouffi C. Louis-Plence P. Bony C. Apparailly F. (2007). Mesenchymal stem cells inhibit the differentiation of dendritic cells through an interleukin-6-dependent mechanism. Stem Cells Dayt. Ohio 25 (8), 2025–2032. 10.1634/stemcells.2006-0548 17510220

[B59] Dou G. Tian R. Liu X. Yuan P. Ye Q. Liu J. (2020). Chimeric apoptotic bodies functionalized with natural membrane and modular delivery system for inflammation modulation. Sci. Adv. 6 (30), eaba2987. 10.1126/sciadv.aba2987 32832662 PMC7439513

[B60] Doyle L. M. Wang M. Z. (2019). Overview of extracellular vesicles, their origin, composition, purpose, and methods for exosome isolation and analysis. Cells 8 (7), 727. 10.3390/cells8070727 31311206 PMC6678302

[B61] Estelles A. Sperinde J. Roulon T. Aguilar B. Bonner C. LePecq J. B. (2007). Exosome nanovesicles displaying G protein-coupled receptors for drug discovery. Int. J. Nanomedicine 2 (4), 751–760. 18203441 PMC2676799

[B62] Favaro E. Carpanetto A. Caorsi C. Giovarelli M. Angelini C. Cavallo-Perin P. (2016). Human mesenchymal stem cells and derived extracellular vesicles induce regulatory dendritic cells in type 1 diabetic patients. Diabetologia 59 (2), 325–333. 10.1007/s00125-015-3808-0 26592240

[B63] Feng K. Xie X. Yuan J. Gong L. Zhu Z. Zhang J. (2021). Reversing the surface charge of MSC-derived small extracellular vesicles by εPL-PEG-DSPE for enhanced osteoarthritis treatment. J. Extracell. Vesicles 10 (13), e12160. 10.1002/jev2.12160 34724347 PMC8559985

[B64] Feng Q. Li Q. Zhou H. Sun L. Lin C. Jin Y. (2023). The role of major immune cells in myocardial infarction. Front. Immunol. 13, 1084460. 10.3389/fimmu.2022.1084460 36741418 PMC9892933

[B65] Forsberg M. H. Kink J. A. Hematti P. Capitini C. M. (2020). Mesenchymal stromal cells and exosomes: progress and challenges. Front. Cell Dev. Biol. 8, 665. 10.3389/fcell.2020.00665 32766255 PMC7379234

[B66] Fraccarollo D. Neuser J. Möller J. Riehle C. Galuppo P. Bauersachs J. (2021). Expansion of CD10neg neutrophils and CD14+HLA-DRneg/low monocytes driving proinflammatory responses in patients with acute myocardial infarction. eLife 10, e66808. 10.7554/elife.66808 34289931 PMC8324297

[B67] Frantz S. Hofmann U. Fraccarollo D. Schäfer A. Kranepuhl S. Hagedorn I. (2013). Monocytes/macrophages prevent healing defects and left ventricular thrombus formation after myocardial infarction. FASEB Journal Official Publication Fed. Am. Soc. Exp. Biol. 27 (3), 871–881. 10.1096/fj.12-214049 23159933

[B68] Frangogiannis N. G. (2014). The inflammatory response in myocardial injury, repair, and remodelling. Nat. Rev. Cardiol. 11 (5), 255–265. 10.1038/nrcardio.2014.28 24663091 PMC4407144

[B69] Fu S. Zhang H. Li X. Zhang Q. Guo C. Qiu K. (2023). Exosomes derived from human amniotic mesenchymal stem cells facilitate diabetic wound healing by angiogenesis and enrich multiple lncRNAs. Tissue Eng. Regen. Med. 20 (2), 295–308. 10.1007/s13770-022-00513-w 36696086 PMC10070558

[B70] Fu S. Wang Z. Huang P. Li G. Niu J. Li Z. (2025). Programmable production of bioactive extracellular vesicles *in vivo* to treat myocardial infarction. Nat. Commun. 16 (1), 2924. 10.1038/s41467-025-58260-0 40133312 PMC11937507

[B71] Gallet R. Dawkins J. Valle J. Simsolo E. de Couto G. Middleton R. (2017). Exosomes secreted by cardiosphere-derived cells reduce scarring, attenuate adverse remodelling, and improve function in acute and chronic porcine myocardial infarction. Eur. Heart J. 38 (3), 201–211. 10.1093/eurheartj/ehw240 28158410 PMC5837390

[B72] Gao L. Qiu F. Cao H. Li H. Dai G. Ma T. (2023). Therapeutic delivery of microRNA-125a-5p oligonucleotides improves recovery from myocardial ischemia/reperfusion injury in mice and swine. Theranostics 13 (2), 685–703. 10.7150/thno.73568 36632217 PMC9830430

[B73] Gupta S. Knowlton A. A. (2007). HSP60 trafficking in adult cardiac myocytes: role of the exosomal pathway. Am. J. Physiology. Heart Circulatory Physiology 292 (6), H3052–H3056. 10.1152/ajpheart.01355.2006 17307989

[B74] Gyöngyösi M. Hemetsberger R. Wolbank S. Pichler V. Kaun C. Posa A. (2011). Delayed recovery of myocardial blood flow after intracoronary stem cell administration. Stem Cell Rev. Rep. 7 (3), 616–623. 10.1007/s12015-010-9213-7 21153508

[B75] Gyöngyösi M. Wojakowski W. Lemarchand P. Lunde K. Tendera M. Bartunek J. (2015). Meta-analysis of cell-based CaRdiac stUdiEs (ACCRUE) in patients with acute myocardial infarction based on individual patient data. Circulation Research 116 (8), 1346–1360. 10.1161/circresaha.116.304346 25700037 PMC4509791

[B76] Gyöngyösi M. Lukovic D. Zlabinger K. Mandic L. Winkler J. Gugerell A. (2017). Cardiac stem cell-based regenerative therapy for the ischemic injured heart — a short update 2017. J. Cardiovasc. Emergencies 3 (2), 81–83. 10.1515/jce-2017-0009

[B77] Herrmann I. K. Wood M. J. A. Fuhrmann G. (2021). Extracellular vesicles as a next-generation drug delivery platform. Nat. Nanotechnol. 16 (7), 748–759. 10.1038/s41565-021-00931-2 34211166

[B78] Hassanzadeh A. Rahman H. S. Markov A. Endjun J. J. Zekiy A. O. Chartrand M. S. (2021). Mesenchymal stem/stromal cell-derived exosomes in regenerative medicine and cancer; overview of development, challenges, and opportunities. Stem Cell Res. and Ther. 12, 297. 10.1186/s13287-021-02378-7 34020704 PMC8138094

[B79] Heijnen H. F. G. Schiel A. E. Fijnheer R. Geuze H. J. Sixma J. J. (1999). Activated platelets release two types of membrane vesicles: microvesicles by surface shedding and exosomes derived from exocytosis of multivesicular bodies and -Granules. Blood 94 (11), 3791–3799. 10.1182/blood.v94.11.3791.423a22_3791_3799 10572093

[B80] Heo J. S. Choi Y. Kim H. O. (2019). Adipose‐Derived Mesenchymal Stem Cells Promote M2 Macrophage Phenotype through Exosomes. Stem Cells Int. 2019, 7921760. 10.1155/2019/7921760 31781246 PMC6875419

[B81] Hilgendorf I. Gerhardt L. M. S. Tan T. C. Winter C. Holderried T. A. W. Chousterman B. G. (2014). Ly-6Chigh monocytes depend on Nr4a1 to balance both inflammatory and reparative phases in the infarcted myocardium. Circulation Research 114 (10), 1611–1622. 10.1161/CIRCRESAHA.114.303204 24625784 PMC4017349

[B82] Hofmann U. Beyersdorf N. Weirather J. Podolskaya A. Bauersachs J. Ertl G. (2012). Activation of CD4+ T lymphocytes improves wound healing and survival after experimental myocardial infarction in mice. Circulation 125 (13), 1652–1663. 10.1161/CIRCULATIONAHA.111.044164 22388323

[B83] Hou M. Wu X. Zhao Z. Deng Q. Chen Y. Yin L. (2022). Endothelial cell-targeting, ROS-ultrasensitive drug/siRNA co-delivery nanocomplexes mitigate early-stage neutrophil recruitment for the anti-inflammatory treatment of myocardial ischemia reperfusion injury. Acta Biomater. 143, 344–355. 10.1016/j.actbio.2022.02.018 35189380

[B84] Hu S. Li Z. Shen D. Zhu D. Huang K. Su T. (2021). Exosome-eluting stents for vascular healing after ischaemic injury. Nat. Biomedical Engineering 5 (10), 1174–1188. 10.1038/s41551-021-00705-0 PMC8490494 33820981

[B85] Huang C. Gu H. Yu Q. Manukyan M. C. Poynter J. A. Wang M. (2011). Sca-1+ cardiac stem cells mediate acute cardioprotection via paracrine factor SDF-1 following myocardial ischemia/reperfusion. PLoS ONE 6 (12), e29246. 10.1371/journal.pone.0029246 22195033 PMC3240662

[B86] Huang F. Zhang J. Zhou H. Qu T. Wang Y. Jiang K. (2024). B cell subsets contribute to myocardial protection by inducing neutrophil apoptosis after ischemia and reperfusion. JCI Insight 9 (4), e167201. 10.1172/jci.insight.167201 38290007 PMC10967377

[B87] Hyvärinen K. Holopainen M. Skirdenko V. Ruhanen H. Lehenkari P. Korhonen M. (2018). Mesenchymal stromal cells and their extracellular vesicles enhance the anti-inflammatory phenotype of regulatory macrophages by downregulating the production of interleukin (IL)-23 and IL-22. Front. Immunol. 9, 771. 10.3389/fimmu.2018.00771 29706969 PMC5906545

[B88] Ibrahim S. A. Khan Y. S. (2025). “Histology, Extracellular Vesicles,” in *Statpearls* (Treasure Island (FL): StatPearls Publishing). 32965927

[B89] Jiang X.-X. Zhang Y. Liu B. Zhang S. X. Wu Y. Yu X. D. (2005). Human mesenchymal stem cells inhibit differentiation and function of monocyte-derived dendritic cells. Blood 105 (10), 4120–4126. 10.1182/blood-2004-02-0586 15692068

[B90] Jiao J. He S. Wang Y. Lu Y. Gu M. Li D. (2021). Regulatory B cells improve ventricular remodeling after myocardial infarction by modulating monocyte migration. Basic Res. Cardiol. 116 (1), 46. 10.1007/s00395-021-00886-4 34302556 PMC8310480

[B91] Ju Z. Ma J. Wang C. Yu J. Qiao Y. Hei F. (2017). Exosomes from iPSCs delivering siRNA attenuate intracellular adhesion Molecule-1 expression and neutrophils adhesion in pulmonary microvascular endothelial cells. Inflammation 40 (2), 486–496. 10.1007/s10753-016-0494-0 28000095

[B92] Kalluri R. LeBleu V. S. (2020). The biology, function, and biomedical applications of exosomes. Science 367 (6478), eaau6977. 10.1126/science.aau6977 32029601 PMC7717626

[B93] Kamerkar S. LeBleu V. S. Sugimoto H. Yang S. Ruivo C. F. Melo S. A. (2017). Exosomes facilitate therapeutic targeting of oncogenic KRAS in pancreatic cancer. Nature 546 (7659), 498–503. 10.1038/nature22341 28607485 PMC5538883

[B94] Khare D. Or R. Resnick I. Barkatz C. Almogi-Hazan O. Avni B. (2018). Mesenchymal stromal cell-derived exosomes affect mRNA expression and function of B-Lymphocytes. Front. Immunol. 9, 3053. 10.3389/fimmu.2018.03053 30622539 PMC6308164

[B95] Kilpinen L. Impola U. Sankkila L. Ritamo I. Aatonen M. Kilpinen S. (2013). Extracellular membrane vesicles from umbilical cord blood-derived MSC protect against ischemic acute kidney injury, a feature that is lost after inflammatory conditioning. J. Extracell. Vesicles 2. 10.3402/jev.v2i0.21927 24349659 PMC3860334

[B96] Kim C. Kim H. Sim W. S. Jung M. Hong J. Moon S. (2024). Spatiotemporal control of neutrophil fate to tune inflammation and repair for myocardial infarction therapy. Nat. Commun. 15 (1), 8481. 10.1038/s41467-024-52812-6 39353987 PMC11445496

[B97] Kooijmans S. A. A. Stremersch S. Braeckmans K. de Smedt S. C. Hendrix A. Wood M. J. A. (2013). Electroporation-induced siRNA precipitation obscures the efficiency of siRNA loading into extracellular vesicles. J. Control. Release Official J. Control. Release Soc. 172 (1), 229–238. 10.1016/j.jconrel.2013.08.014 23994516

[B98] Kordelas L. Schwich E. Dittrich R. Horn P. A. Beelen D. W. Börger V. (2019). Individual immune-modulatory capabilities of MSC-derived extracellular vesicle (EV) preparations and recipient-dependent responsiveness. Int. J. Mol. Sci. 20 (7), 1642. 10.3390/ijms20071642 30987036 PMC6479947

[B99] Kosaka N. Yoshioka Y. Fujita Y. Ochiya T. (2016). Versatile roles of extracellular vesicles in cancer. J. Clin. Investigation 126 (4), 1163–1172. 10.1172/JCI81130 26974161 PMC4811151

[B100] Kou M. Huang L. Yang J. Chiang Z. Chen S. Liu J. (2022). Mesenchymal stem cell-derived extracellular vesicles for immunomodulation and regeneration: a next generation therapeutic tool? Cell Death and Dis. 13 (7), 580. 10.1038/s41419-022-05034-x PMC9252569 35787632

[B101] Kubota A. Suto A. Suga K. Iwata A. Tanaka S. Suzuki K. (2021). Inhibition of Interleukin-21 prolongs the survival through the promotion of wound healing after myocardial infarction. J. Mol. Cell. Cardiol. 159, 48–61. 10.1016/j.yjmcc.2021.06.006 34144051

[B102] Lai R. C. Arslan F. Lee M. M. Sze N. S. K. Choo A. Chen T. S. (2010). Exosome secreted by MSC reduces myocardial ischemia/reperfusion injury. Stem Cell Res. 4 (3), 214–222. 10.1016/j.scr.2009.12.003 20138817

[B103] Lang J. K. Young R. F. Ashraf H. Canty J. M. (2016). Inhibiting extracellular vesicle release from human cardiosphere derived cells with lentiviral knockdown of nSMase2 differentially effects proliferation and apoptosis in cardiomyocytes, fibroblasts and endothelial cells *in vitro* . PLoS ONE 11 (11), e0165926. 10.1371/journal.pone.0165926 27806113 PMC5091915

[B104] Lazar E. Benedek T. Korodi S. Rat N. Lo J. Benedek I. (2018). Stem cell-derived exosomes - an emerging tool for myocardial regeneration. World J. Stem Cells 10 (8), 106–115. 10.4252/wjsc.v10.i8.106 30190780 PMC6121000

[B105] Leistner D. M. Fischer-Rasokat U. Honold J. Seeger F. H. Schächinger V. Lehmann R. (2011). Transplantation of progenitor cells and regeneration enhancement in acute myocardial infarction (TOPCARE-AMI): final 5-year results suggest long-term safety and efficacy. Clin. Res. Cardiol. Official J. Ger. Cardiac Soc. 100 (10), 925–934. 10.1007/s00392-011-0327-y 21633921

[B106] Lener T. Gimona M. Aigner L. Börger V. Buzas E. Camussi G. (2015). Applying Extracellular Vesicles Based Therapeutics in Clinical Trials - an ISEV Position Paper. J. Extracell Vesicles 4 (10), 30087. 10.3402/jev.v4.30087 26725829 PMC4698466

[B107] Leor J. Rozen L. Zuloff-Shani A. Feinberg M. S. Amsalem Y. Barbash I. M. (2006). *Ex vivo* activated human macrophages improve healing, remodeling, and function of the infarcted heart. Circulation 114 (1), 94–100. 10.1161/CIRCULATIONAHA.105.000331 16820652

[B108] Li J. Yang K. Y. Tam R. C. Y. Chan V. W. Lan H. Y. Hori S. (2019). Regulatory T-cells regulate neonatal heart regeneration by potentiating cardiomyocyte proliferation in a paracrine manner. Theranostics 9 (15), 4324–4341. 10.7150/thno.32734 31285764 PMC6599663

[B109] Li J. Liang C. Yang K. Y. Huang X. Han M. Y. Li X. (2020). Specific ablation of CD4+ T-cells promotes heart regeneration in juvenile mice. Theranostics 10 (18), 8018–8035. 10.7150/thno.42943 32724455 PMC7381734

[B110] Li Q. Huang Z. Wang Q. Gao J. Chen J. Tan H. (2022). Targeted immunomodulation therapy for cardiac repair by platelet membrane engineering extracellular vesicles via hitching peripheral monocytes. Biomaterials 284, 121529. 10.1016/j.biomaterials.2022.121529 35447403

[B111] Li S. Yang K. Cao W. Guo R. Liu Z. Zhang J. (2023). Tanshinone IIA enhances the therapeutic efficacy of mesenchymal stem cells derived exosomes in myocardial ischemia/reperfusion injury via up-regulating miR-223-5p. J. Control. Release Official J. Control. Release Soc. 358, 13–26. 10.1016/j.jconrel.2023.04.014 37086952

[B112] Li J. Sun S. Zhu D. Mei X. Lyu Y. Huang K. (2024). Inhalable stem cell exosomes promote heart repair post-myocardial infarction. Circulation 150 (9), 710–723. 10.1161/circulationaha.123.065005 39186525 PMC11349039

[B113] Li X. Li Z. Liu J. Zang H. Guo S. Yang Y. (2026). Engineering stem cell-based nanotherapeutics to overcome myocardial ischemia-reperfusion injury. Biomaterials 331, 124121. 10.1016/j.biomaterials.2026.124121 41831416

[B114] Lightner A. L. Sengupta V. Qian S. Ransom J. T. Suzuki S. Park D. J. (2023). Bone marrow mesenchymal stem cell derived extracellular vesicle infusion for the treatment of respiratory failure from COVID-19: a randomized placebo controlled dosing clinical trial. Chest 164 (6), 1444–1453. 10.1016/j.chest.2023.06.024 37356708 PMC10289818

[B115] Lin Y.-z. Wu B. w. Lu Z. d. Huang Y. Shi Y. Liu H. (2013). Circulating Th22 and Th9 levels in patients with acute coronary syndrome. Mediat. Inflamm. 2013, 635672. 10.1155/2013/635672 24453425 PMC3884785

[B116] Linke A. Müller P. Nurzynska D. Casarsa C. Torella D. Nascimbene A. (2005). Stem cells in the dog heart are self-renewing, clonogenic, and multipotent and regenerate infarcted myocardium, improving cardiac function. Proc. Natl. Acad. Sci. U. S. A. 102 (25), 8966–8971. 10.1073/pnas.0502678102 15951423 PMC1157041

[B117] Liu L. Wang Y. Fan H. Zhao X. Liu D. Hu Y. (2012). MicroRNA-181a regulates local immune balance by inhibiting proliferation and immunosuppressive properties of mesenchymal stem cells. Stem Cells Dayt. Ohio 30 (8), 1756–1770. 10.1002/stem.1156 22714950

[B118] Liu Y. Yin Z. Zhang R. Yan K. Chen L. Chen F. (2014). MSCs inhibit bone marrow-derived DC maturation and function through the release of TSG-6. Biochem. Biophysical Res. Commun. 450 (4), 1409–1415. 10.1016/j.bbrc.2014.07.001 25014173

[B119] Liu H. Gao W. Yuan J. Wu C. Yao K. Zhang L. (2016). Exosomes derived from dendritic cells improve cardiac function via activation of CD4+ T lymphocytes after myocardial infarction. J. Mol. Cell. Cardiol. 91, 123–133. 10.1016/j.yjmcc.2015.12.028 26746143

[B120] Liu J. J. Wang H. Li J. (2016). Inflammation and Inflammatory Cells in Myocardial Infarction and Reperfusion Injury: A Double-Edged Sword. Clin Med Insights Cardiol 10:79-84. 10.4137/CMC.S33164 27279755 PMC4892199

[B121] Liu B. Lee B. W. Nakanishi K. Villasante A. Williamson R. Metz J. (2018). Cardiac recovery via extended cell-free delivery of extracellular vesicles secreted by cardiomyocytes derived from induced pluripotent stem cells. Nat. Biomedical Engineering 2 (5), 293–303. 10.1038/s41551-018-0229-7 30271672 PMC6159913

[B122] Liu H. Sun X. Gong X. Wang G. (2019). Human umbilical cord mesenchymal stem cells derived exosomes exert antiapoptosis effect via activating PI3K/Akt/mTOR pathway on H9C2 cells. J. Cell. Biochem. 120 (9), 14455–14464. 10.1002/jcb.28705 30989714

[B123] Liu J. Liu F. Liang T. Zhou Y. Su X. Li X. (2024). The roles of Th cells in myocardial infarction. Cell Death Discov. 10 (1), 287. 10.1038/s41420-024-02064-6 38879568 PMC11180143

[B124] Lo Sicco C. Reverberi D. Balbi C. Ulivi V. Principi E. Pascucci L. (2017). Mesenchymal stem cell‐derived extracellular vesicles as mediators of anti‐inflammatory effects: endorsement of macrophage polarization. Stem Cells Transl. Med. 6 (3), 1018–1028. 10.1002/sctm.16-0363 28186708 PMC5442783

[B125] Loyer X. Zlatanova I. Devue C. Yin M. Howangyin K. Y. Klaihmon P. (2018). Intra-cardiac release of extracellular vesicles shapes inflammation following myocardial infarction. Circulation Res. 123 (1), 100–106. 10.1161/CIRCRESAHA.117.311326 29592957 PMC6023578

[B126] Ma Y. (2021). Role of neutrophils in cardiac injury and repair following myocardial infarction. Cells 10 (7), 1676. 10.3390/cells10071676 34359844 PMC8305164

[B127] Ma Y. Yabluchanskiy A. Iyer R. P. Cannon P. L. Flynn E. R. Jung M. (2016). Temporal neutrophil polarization following myocardial infarction. Cardiovasc. Res. 110 (1), 51–61. 10.1093/cvr/cvw024 26825554 PMC4798046

[B128] Ma Q. Fan Q. Han X. Dong Z. Xu J. Bai J. (2021). Platelet-derived extracellular vesicles to target plaque inflammation for effective anti-atherosclerotic therapy. J. Control. Release 329, 445–453. 10.1016/j.jconrel.2020.11.064 33285103

[B129] Maas S. L. N. Breakefield X. O. Weaver A. M. (2017). Extracellular vesicles: unique intercellular delivery vehicles. Trends Cell Biol. 27 (3), 172–188. 10.1016/j.tcb.2016.11.003 27979573 PMC5318253

[B130] Madel R. J. Börger V. Dittrich R. Bremer M. Tertel T. Phuong N. N. T. (2023). Independent human mesenchymal stromal cell-derived extracellular vesicle preparations differentially attenuate symptoms in an advanced murine graft-versus-host disease model. Cytotherapy 25 (8), 821–836. 10.1016/j.jcyt.2023.03.008 37055321

[B131] Madonna R. Van Laake L. W. Davidson S. M Engel F. B. Hausenloy D. J. Lecour S. (2016). Position paper of the european society of cardiology working group cellular biology of the heart: cell-based therapies for myocardial repair and regeneration in ischemic heart disease and heart failure. Eur. Heart Journal 37 (23), 1789–1798. 10.1093/eurheartj/ehw113 27055812 PMC4912026

[B132] Maleki B. Alani B. Tamehri Zadeh S. S. Saadat S. Rajabi A. Ayoubzadeh S. M. J. (2022). MicroRNAs and exosomes: cardiac stem cells in heart diseases. Pathology, Res. Pract. 229, 153701. 10.1016/j.prp.2021.153701 34872024

[B133] Maring J. A. Lodder K. Mol E. Verhage V. Wiesmeijer K. C. Dingenouts C. K. E. (2019). Cardiac progenitor cell–derived extracellular vesicles reduce infarct size and associate with increased cardiovascular cell proliferation. J. Cardiovasc. Transl. Res. 12 (1), 5–17. 10.1007/s12265-018-9842-9 30456736 PMC6394631

[B134] Marinković G. Grauen Larsen H. Yndigegn T. Szabo I. A. Mares R. G. de Camp L. (2019). Inhibition of pro-inflammatory myeloid cell responses by short-term S100A9 blockade improves cardiac function after myocardial infarction. Eur. Heart J. 40 (32), 2713–2723. 10.1093/eurheartj/ehz461 31292614

[B135] Matsumoto K. Ogawa M. Suzuki J. i. Hirata Y. Nagai R. Isobe M. (2011). Regulatory T lymphocytes attenuate myocardial infarction-induced ventricular remodeling in mice. Int. Heart J. 52 (6), 382–387. 10.1536/ihj.52.382 22188713

[B136] Menard C. Pacelli L. Bassi G. Dulong J. Bifari F. Bezier I. (2013). Clinical-grade mesenchymal stromal cells produced under various good manufacturing practice processes differ in their immunomodulatory properties: standardization of immune quality controls. Stem Cells Dev. 22 (12), 1789–1801. 10.1089/scd.2012.0594 23339531 PMC3668498

[B137] Mokarizadeh A. Delirezh N. Morshedi A. Mosayebi G. Farshid A. A. Mardani K. (2012). Microvesicles derived from mesenchymal stem cells: potent organelles for induction of tolerogenic signaling. Immunol. Lett. 147 (1-2), 47–54. 10.1016/j.imlet.2012.06.001 22705267

[B138] Nagai T. Honda S. Sugano Y. Matsuyama T. Ohta‐Ogo K. Asaumi Y. (2014). Decreased myocardial dendritic cells is associated with impaired reparative fibrosis and development of cardiac rupture after myocardial infarction in humans. J. Am. Heart Assoc. Cardiovasc. Cerebrovasc. Dis. 3 (3), e000839. 10.1161/jaha.114.000839 24895162 PMC4309075

[B139] Nahrendorf M. Swirski F. K. Aikawa E. Stangenberg L. Wurdinger T. Figueiredo J. L. (2007). The healing myocardium sequentially mobilizes two monocyte subsets with divergent and complementary functions. J. Exp. Med. 204 (12), 3037–3047. 10.1084/jem.20070885 18025128 PMC2118517

[B140] Nojehdehi S. Soudi S. Hesampour A. Rasouli S. Soleimani M. Hashemi S. M. (2018). Immunomodulatory effects of mesenchymal stem cell-derived exosomes on experimental type-1 autoimmune diabetes. J. Cell. Biochem. 119 (11), 9433–9443. 10.1002/jcb.27260 30074271

[B141] Okoye I. S. Coomes S. M. Pelly V. S. Czieso S. Papayannopoulos V. Tolmachova T. (2014). MicroRNA-Containing T-Regulatory-Cell-Derived exosomes suppress pathogenic T helper 1 cells. Immunity 41 (1), 89–103. 10.1016/j.immuni.2014.08.008 25035954 PMC4104030

[B142] Ong S.-B. Hernández-Reséndiz S. Crespo-Avilan G. E. Mukhametshina R. T. Kwek X. Y. Cabrera-Fuentes H. A. (2018). Inflammation following acute myocardial infarction: multiple players, dynamic roles, and novel therapeutic opportunities. Pharmacol. and Ther. 186, 73–87. 10.1016/j.pharmthera.2018.01.001 29330085 PMC5981007

[B143] Pan Y. Li L. Cao N. Liao J. Chen H. Zhang M. (2025). Advanced nano delivery system for stem cell therapy for alzheimer's disease. Biomaterials 314, 122852. 10.1016/j.biomaterials.2024.122852 39357149

[B144] Pegtel D. M. Cosmopoulos K. Thorley-Lawson D. A. van Eijndhoven M. A. J. Hopmans E. S. Lindenberg J. L. (2010). Functional delivery of viral miRNAs via exosomes. Proc. Natl. Acad. Sci. U. S. A. 107 (14), 6328–6333. 10.1073/pnas.0914843107 20304794 PMC2851954

[B145] Peng W. Yang Y. Chen J. Xu Z. Lou Y. Li Q. (2023). Small extracellular vesicles secreted by iPSC-Derived MSCs ameliorate pulmonary inflammation and lung injury induced by sepsis through delivery of miR-125b-5p. J. Immunol. Res. 2023, 8987049. 10.1155/2023/8987049 37425491 PMC10329558

[B146] Pezzana C. Agnely F. Bochot A. Siepmann J. Menasché P. (2021). Extracellular vesicles and biomaterial design: new therapies for cardiac repair. Trends Mol. Med. 27 (3), 231–247. 10.1016/j.molmed.2020.10.006 33218944

[B147] Pironti G. Strachan R. T. Abraham D. Mon-Wei Yu S. Chen M. Chen W. (2015). Circulating exosomes induced by cardiac pressure overload contain functional angiotensin II type 1 receptors. Circulation 131 (24), 2120–2130. 10.1161/CIRCULATIONAHA.115.015687 25995315 PMC4470842

[B148] Popowski K. D. López de Juan Abad B. George A. Silkstone D. Belcher E. Chung J. (2022). Inhalable exosomes outperform liposomes as mRNA and protein drug carriers to the lung. Extracell. Vesicle 1, 100002. 10.1016/j.vesic.2022.100002 36523538 PMC9213043

[B149] Puhl S.-L. Steffens S. (2019). Neutrophils in post-myocardial infarction inflammation: damage vs. resolution? Front. Cardiovasc. Med. 6, 25. 10.3389/fcvm.2019.00025 30937305 PMC6431642

[B150] Rafei M. Campeau P. M. Aguilar-Mahecha A. Buchanan M. Williams P. Birman E. (2009). Mesenchymal stromal cells ameliorate experimental autoimmune encephalomyelitis by inhibiting CD4 Th17 T cells in a CC chemokine ligand 2-dependent manner. J. Immunol. Baltim. Md. 1950 182 (10), 5994–6002. 10.4049/jimmunol.0803962 19414750

[B151] Ramos Y. F. M. Tertel T. Shaw G. Staubach S. de Almeida R. C. Suchiman E. (2022). Characterizing the secretome of licensed hiPSC-derived MSCs. Stem Cell Res. and Ther. 13, 434. 10.1186/s13287-022-03117-2 36056373 PMC9438242

[B152] Reis M. Mavin E. Nicholson L. Green K. Dickinson A. M. Wang X. N. (2018). Mesenchymal stromal cell-derived extracellular vesicles attenuate dendritic cell maturation and function. Front. Immunol. 9, 2538. 10.3389/fimmu.2018.02538 30473695 PMC6237916

[B153] Rocha-Resende C. Pani F. Adamo L. (2021). B cells modulate the expression of MHC-II on cardiac CCR2− macrophages. J. Molecular Cellular Cardiology 157, 98–103. 10.1016/j.yjmcc.2021.05.003 PMC8319082 33971183

[B154] Rohani C. Jafarpoor H. Mortazavi Y. Esbakian B. Gholinia H. (2022). Mortality in patients with myocardial infarction and potential risk factors: a five-year data analysis. ARYA Atheroscler. 18 (3), 1–8. 10.48305/arya.v18i0.2427 36815954 PMC9931944

[B155] Sager H. B. Hulsmans M. Lavine K. J. Moreira M. B. Heidt T. Courties G. (2016). Proliferation and recruitment contribute to myocardial macrophage expansion in chronic heart failure. Circulation Res. 119 (7), 853–864. 10.1161/CIRCRESAHA.116.309001 27444755 PMC5378496

[B156] Santos-Zas I. Lemarié J. Zlatanova I. Cachanado M. Seghezzi J. C. Benamer H. (2021). Cytotoxic CD8+ T cells promote granzyme B-dependent adverse post-ischemic cardiac remodeling. Nat. Commun. 12, 1483. 10.1038/s41467-021-21737-9 33674611 PMC7935973

[B157] Santoso M. R. Ikeda G. Tada Y. Jung J. H. Vaskova E. Sierra R. G. (2020). Exosomes from induced pluripotent stem cell–derived cardiomyocytes promote autophagy for myocardial repair. J. Am. Heart Assoc. Cardiovasc. Cerebrovasc. Dis. 9 (6), e014345. 10.1161/JAHA.119.014345 32131688 PMC7335524

[B158] Selvasandran K. Makhoul G. Jaiswal P. K. Jurakhan R. Li L. Ridwan K. (2018). A tumor necrosis Factor-α and hypoxia-induced secretome therapy for myocardial repair. Ann. Thorac. Surg. 105 (3), 715–723. 10.1016/j.athoracsur.2017.09.005 29258676

[B159] Sha Z. Liu W. Jiang T. Zhang K. Yu Z. (2024). Astragaloside IV induces the protective effect of bone marrow mesenchymal stem cells derived exosomes in acute myocardial infarction by inducing angiogenesis and inhibiting apoptosis. Biotechnol. and Genet. Eng. Rev. 40 (3), 1438–1455. 10.1080/02648725.2023.2194087 36971224

[B160] Shahir M. Mahmoud Hashemi S. Asadirad A. Varahram M. Kazempour-Dizaji M. Folkerts G. (2020). Effect of mesenchymal stem cell‐derived exosomes on the induction of mouse tolerogenic dendritic cells. J. Cell. Physiology 235 (10), 7043–7055. 10.1002/jcp.29601 32043593 PMC7496360

[B161] Shao L. Zhang Y. Lan B. Wang J. Zhang Z. Zhang L. (2017). MiRNA-Sequence indicates that mesenchymal stem cells and exosomes have similar mechanism to enhance cardiac repair. BioMed Res. Int. 2017, 4150705. 10.1155/2017/4150705 28203568 PMC5292186

[B162] Sharir R. Semo J. Shimoni S. Ben-Mordechai T. Landa-Rouben N. Maysel-Auslender S. (2014). Experimental myocardial infarction induces altered regulatory T cell hemostasis, and adoptive transfer attenuates subsequent remodeling. PLoS ONE 9 (12), e113653. 10.1371/journal.pone.0113653 25436994 PMC4249913

[B163] Skorska A. von Haehling S. Ludwig M. Lux C. A. Gaebel R. Kleiner G. (2015). The CD4+AT2R+ T cell subpopulation improves post-infarction remodelling and restores cardiac function. J. Cell. Mol. Med. 19 (8), 1975–1985. 10.1111/jcmm.12574 25991381 PMC4549048

[B164] Song Y. Dou H. Li X. Zhao X. Li Y. Liu D. (2017). Exosomal miR-146a contributes to the enhanced therapeutic efficacy of Interleukin-1β-Primed mesenchymal stem cells against sepsis. Stem Cells Dayt. Ohio 35 (5), 1208–1221. 10.1002/stem.2564 28090688

[B165] Song Y. Huang Z. Liu X. Pang Z. Chen J. Yang H. (2019). Platelet membrane-coated nanoparticle-mediated targeting delivery of rapamycin blocks atherosclerotic plaque development and stabilizes plaque in apolipoprotein E-deficient (ApoE−/−) mice. Nanomedicine Nanotechnol. Biol. Med. 15 (1), 13–24. 10.1016/j.nano.2018.08.002 30171903

[B166] Stuffers S. Wegner C. S. Stenmark H. Brech A. (2009). Multivesicular endosome biogenesis in the absence of ESCRTs. Traffic. 10(7):925-937. 10.1111/j.1600-0854.2009.00920.x 19490536

[B167] Sun X. Shan A. Wei Z. Xu B. (2018). Intravenous mesenchymal stem cell-derived exosomes ameliorate myocardial inflammation in the dilated cardiomyopathy. Biochem. Biophysical Res. Commun. 503 (4), 2611–2618. 10.1016/j.bbrc.2018.08.012 30126637

[B168] Sun Y. Pinto C. Camus S. Duval V. Alayrac P. Zlatanova I. (2022). Splenic marginal zone B lymphocytes regulate cardiac remodeling after acute myocardial infarction in mice. J. Am. Coll. Cardiol. 79 (7), 632–647. 10.1016/j.jacc.2021.11.051 35177192

[B169] Tachibana A. Santoso M. R. Mahmoudi M. Shukla P. Wang L. Bennett M. (2017). Paracrine effects of the pluripotent stem cell-derived cardiac myocytes salvage the injured myocardium. Circulation Research 121 (6), e22–e36. 10.1161/CIRCRESAHA.117.310803 28743804 PMC5783162

[B170] Takakura Y. Hanayama R. Akiyoshi K. Futaki S. Hida K. Ichiki T. (2024). Quality and safety considerations for therapeutic products based on extracellular vesicles. Pharm. Res. 41 (8), 1573–1594. 10.1007/s11095-024-03757-4 39112776 PMC11362369

[B171] Tang T.-T. Yuan J. Zhu Z. F. Zhang W. C. Xiao H. Xia N. (2012). Regulatory T cells ameliorate cardiac remodeling after myocardial infarction. Basic Res. Cardiol. 107 (1), 232. 10.1007/s00395-011-0232-6 22189560

[B172] Tang T.-T. Li Y. Y. Li J. J. Wang K. Han Y. Dong W. Y. (2018). Liver-heart crosstalk controls IL-22 activity in cardiac protection after myocardial infarction. Theranostics 8 (16), 4552–4562. 10.7150/thno.24723 30214638 PMC6134935

[B173] Tertel T. Dittrich R. Arsène P. Jensen A. Giebel B. (2023). EV products obtained from iPSC-derived MSCs show batch-to-batch variations in their ability to modulate allogeneic immune responses *in vitro* . Front. Cell Dev. Biol. 11, 1282860. 10.3389/fcell.2023.1282860 37965578 PMC10642442

[B174] Théry C. Witwer K. W. Aikawa E. Alcaraz M. J. Anderson J. D. Andriantsitohaina R. (2018). Minimal Information for Studies of Extracellular Vesicles 2018 (MISEV2018): A Position Statement of the International Society for Extracellular Vesicles and Update of the MISEV2014 Guidelines. J. Extracell Vesicles 7 (1):1535750. 10.1080/20013078.2018.1535750 30637094 PMC6322352

[B175] Ti D. Hao H. Tong C. Liu J. Dong L. Zheng J. (2015). LPS-preconditioned mesenchymal stromal cells modify macrophage polarization for resolution of chronic inflammation via exosome-shuttled let-7b. J. Transl. Med. 13, 308. 10.1186/s12967-015-0642-6 26386558 PMC4575470

[B176] Tian T. Zhang H. X. He C. P. Fan S. Zhu Y. L. Qi C. (2018). Surface functionalized exosomes as targeted drug delivery vehicles for cerebral ischemia therapy. Biomaterials 150, 137–149. 10.1016/j.biomaterials.2017.10.012 29040874

[B177] Timmers L. Lim S. K. Hoefer I. E. Arslan F. Lai R. C. van Oorschot A. A. M. (2011). Human mesenchymal stem cell-conditioned medium improves cardiac function following myocardial infarction. Stem Cell Res. 6 (3), 206–214. 10.1016/j.scr.2011.01.001 21419744

[B178] Tkach M. Théry C. (2016). Communication by extracellular vesicles: where we are and where we need to Go. Cell 164 (6), 1226–1232. 10.1016/j.cell.2016.01.043 26967288

[B179] Toh W. S. Lai R. C. Hui J. H. P. Lim S. K. (2017). MSC exosome as a cell-free MSC therapy for cartilage regeneration: implications for osteoarthritis treatment. Seminars Cell and Dev. Biol. 67, 56–64. 10.1016/j.semcdb.2016.11.008 27871993

[B180] Tomasoni S. Longaretti L. Rota C. Morigi M. Conti S. Gotti E. (2013). Transfer of growth factor receptor mRNA Via exosomes unravels the regenerative effect of mesenchymal stem cells. Stem Cells Dev. 22 (5), 772–780. 10.1089/scd.2012.0266 23082760 PMC3578372

[B181] Vagnozzi R. J. Maillet M. Sargent M. A. Khalil H. Johansen A. K. Z. Schwanekamp J. A. (2020). An acute immune response underlies the benefit of cardiac stem cell therapy. Nature 577 (7790), 405–409. 10.1038/s41586-019-1802-2 31775156 PMC6962570

[B182] van Berlo J. H. Kanisicak O. Maillet M. Vagnozzi R. J. Karch J. Lin S. C. J. (2014). c-kit+ cells minimally contribute cardiomyocytes to the heart. Nature 509 (7500), 337–341. 10.1038/nature13309 24805242 PMC4127035

[B183] Van Delen M. Derdelinckx J. Wouters K. Nelissen I. Cools N. (2024). A systematic review and meta-analysis of clinical trials assessing safety and efficacy of human extracellular vesicle-based therapy. J. Extracell. Vesicles 13 (7), e12458. 10.1002/jev2.12458 38958077 PMC11220457

[B184] Van der Borght K. Scott C. L. Nindl V. Bouché A. Martens L. Sichien D. (2017). Myocardial infarction primes autoreactive T cells through activation of dendritic cells. Cell Rep. 18 (12), 3005–3017. 10.1016/j.celrep.2017.02.079 28329691 PMC5379012

[B185] Vaskova E. Ikeda G. Tada Y. Wahlquist C. Mercola M. Yang P. C. (2020). Sacubitril/Valsartan improves cardiac function and decreases myocardial fibrosis Via downregulation of exosomal miR-181a in a rodent chronic myocardial infarction model. J. Am. Heart Assoc. 9 (13), e015640. 10.1161/JAHA.119.015640 32538237 PMC7670523

[B186] Vrijsen K. R. Sluijter J. P. G. Schuchardt M. W. L. van Balkom B. W. M. Noort W. A. Chamuleau S. A. J. (2010). Cardiomyocyte progenitor cell-derived exosomes stimulate migration of endothelial cells. J. Cell. Mol. Med. 14 (5), 1064–1070. 10.1111/j.1582-4934.2010.01081.x 20465578 PMC3822742

[B187] Wang X. Ha T. Zou J. Ren D. Liu L. Zhang X. (2014). MicroRNA-125b protects against myocardial ischaemia/reperfusion injury via targeting p53-mediated apoptotic signalling and TRAF6. Cardiovasc. Res. 102 (3), 385–395. 10.1093/cvr/cvu044 24576954 PMC4030511

[B188] Wang K. Wen S. Jiao J. Tang T. Zhao X. Zhang M. (2018). IL-21 promotes myocardial ischaemia/reperfusion injury through the modulation of neutrophil infiltration. Br. J. Pharmacol. 175 (8), 1329–1343. 10.1111/bph.13781 28294304 PMC5866974

[B189] Wang Y. Zhao R. Liu D. Deng W. Xu G. Liu W. (2018). Exosomes derived from miR-214-Enriched bone marrow-derived mesenchymal stem cells regulate oxidative damage in cardiac stem cells by targeting CaMKII. Oxidative Med. Cell. Longev. 2018, 4971261. 10.1155/2018/4971261 30159114 PMC6109555

[B190] Wang J. Liu M. Wu Q. Li Q. Gao L. Jiang Y. (2019). Human embryonic stem cell-derived cardiovascular progenitors repair infarcted hearts through modulation of macrophages via activation of signal transducer and activator of transcription 6. Antioxidants and Redox Signal. 31 (5), 369–386. 10.1089/ars.2018.7688 30854870 PMC6602123

[B191] Wang X. Hu S. Li J. Zhu D. Wang Z. Cores J. (2021). Extruded mesenchymal stem cell nanovesicles are equally potent to natural extracellular vesicles in cardiac repair. ACS Applied Materials and Interfaces 13 (47), 55767–55779. 10.1021/acsami.1c08044 34793116

[B192] Wei Z. Qiao S. Zhao J. Liu Y. Li Q. Wei Z. (2019). miRNA-181a over-expression in mesenchymal stem cell-derived exosomes influenced inflammatory response after myocardial ischemia-reperfusion injury. Life Sci. 232, 116632. 10.1016/j.lfs.2019.116632 31278944

[B193] Weirather J. Hofmann U. D. W. Beyersdorf N. Ramos G. C. Vogel B. Frey A. (2014). Foxp3+ CD4+ T cells improve healing after myocardial infarction by modulating monocyte/macrophage differentiation. Circulation Res. 115 (1), 55–67. 10.1161/CIRCRESAHA.115.303895 24786398

[B194] Wollert K. C. Meyer G. P. Lotz J. Ringes-Lichtenberg S. Lippolt P. Breidenbach C. (2004). Intracoronary autologous bone-marrow cell transfer after myocardial infarction: the BOOST randomised controlled clinical trial. Lancet London, Engl. 364 (9429), 141–148. 10.1016/S0140-6736(04)16626-9 15246726

[B195] Wu L. Dalal R. Cao C. D. Postoak J. L. Yang G. Zhang Q. (2019). IL-10-producing B cells are enriched in murine pericardial adipose tissues and ameliorate the outcome of acute myocardial infarction. Proc. Natl. Acad. Sci. U. S. A. 116 (43), 21673–21684. 10.1073/pnas.1911464116 31591231 PMC6815157

[B196] Xiao J. Yang R. Biswas S. Qin X. Zhang M. Deng W. (2015). Mesenchymal stem cells and induced pluripotent stem cells as therapies for multiple sclerosis. Int. Journal Molecular Sciences 16 (5), 9283–9302. 10.3390/ijms16059283 25918935 PMC4463588

[B197] Xu R. Zhang F. Chai R. Zhou W. Hu M. Liu B. (2019). Exosomes derived from pro‐inflammatory bone marrow‐derived mesenchymal stem cells reduce inflammation and myocardial injury via mediating macrophage polarization. J. Cell. Mol. Med. 23 (11), 7617–7631. 10.1111/jcmm.14635 31557396 PMC6815833

[B198] Xu Y. Jiang K. Chen F. Qian J. Wang D. Wu Y. (2022). Bone marrow-derived naïve B lymphocytes improve heart function after myocardial infarction: a novel cardioprotective mechanism for empagliflozin. Basic Research Cardiology 117 (1), 47. 10.1007/s00395-022-00956-1 36171393

[B199] Xue J. Ge H. Lin Z. Wang H. Lin W. Liu Y. (2019). The role of dendritic cells regulated by HMGB1/TLR4 signalling pathway in myocardial ischaemia reperfusion injury. J. Cell. Mol. Med. 23 (4), 2849–2862. 10.1111/jcmm.14192 30784177 PMC6433676

[B200] Yan X. Anzai A. Katsumata Y. Matsuhashi T. Ito K. Endo J. (2013). Temporal dynamics of cardiac immune cell accumulation following acute myocardial infarction. J. Mol. Cell. Cardiol. 62, 24–35. 10.1016/j.yjmcc.2013.04.023 23644221

[B201] Yáñez-Mó M. Siljander P. R-M. Andreu Z. Zavec A. B. Borràs F. E. Buzas E. I (2015). Biological Properties of Extracellular Vesicles and their Physiological Functions. J. Extracell Vesicles.4 27066. 10.3402/jev.v4.27066 25979354 PMC4433489

[B202] Ye T. Chen Z. Zhang J. Luo L. Gao R. Gong L. (2023). Large extracellular vesicles secreted by human iPSC-derived MSCs ameliorate tendinopathy via regulating macrophage heterogeneity. Bioact. Mater. 21, 194–208. 10.1016/j.bioactmat.2022.08.007 36101856 PMC9440485

[B203] Yellon D. M. Davidson S. M. (2014). Exosomes: nanoparticles involved in cardioprotection? Circulation Res. 114 (2), 325–332. 10.1161/CIRCRESAHA.113.300636 24436428

[B204] Yu B. Gong M. Wang Y. Millard R. W. Pasha Z. Yang Y. (2013a). Cardiomyocyte protection by GATA-4 gene engineered mesenchymal stem cells is partially mediated by translocation of miR-221 in microvesicles. PLoS ONE 8 (8), e73304. 10.1371/journal.pone.0073304 24015301 PMC3756018

[B205] Yu B. Gong M. He Z. Wang Y. G. Millard R. W. Ashraf M. (2013b). Enhanced mesenchymal stem cell survival induced by GATA-4 overexpression is partially mediated by regulation of the miR-15 family. International Journal Biochemistry and Cell Biology 45 (12), 2724–2735. 10.1016/j.biocel.2013.09.007 24070634 PMC3863644

[B206] Zeng Z. Yu K. Chen L. Li W. Xiao H. Huang Z. (2016). Interleukin-2/Anti-Interleukin-2 immune complex attenuates cardiac remodeling after myocardial infarction through expansion of regulatory T cells. J. Immunol. Res. 2016, 8493767. 10.1155/2016/8493767 27144181 PMC4837274

[B207] Zhang Z. Yang J. Yan W. Li Y. Shen Z. Asahara T. (2016). Pretreatment of cardiac stem cells with exosomes derived from mesenchymal stem cells enhances myocardial repair. J. Am. Heart Assoc. Cardiovasc. Cerebrovasc. Dis. 5 (1), e002856. 10.1161/JAHA.115.002856 26811168 PMC4859399

[B208] Zhang X. Liu S. Weng X. Zeng S. Yu L. Guo J. (2018). Brg1 deficiency in vascular endothelial cells blocks neutrophil recruitment and ameliorates cardiac ischemia-reperfusion injury in mice. Int. J. Cardiol. 269, 250–258. 10.1016/j.ijcard.2018.07.105 30049497

[B209] Zhang B. Yeo R. W. Y. Lai R. C. Sim E. W. K. Chin K. C. Lim S. K. (2018). Mesenchymal stromal cell exosome-enhanced regulatory T-cell production through an antigen-presenting cell-mediated pathway. Cytotherapy 20 (5), 687–696. 10.1016/j.jcyt.2018.02.372 29622483

[B210] Zhang Q. Fu L. Liang Y. Guo Z. Wang L. Ma C. (2018). Exosomes originating from MSCs stimulated with TGF-β and IFN-γ promote treg differentiation. J. Cell. Physiology 233 (9), 6832–6840. 10.1002/jcp.26436 29336475 PMC13482522

[B211] Zhang Z. Tian H. Yang C. Liu J. Zhang H. Wang J. (2020). Mesenchymal stem cells promote the resolution of cardiac inflammation after ischemia reperfusion Via enhancing efferocytosis of neutrophils. J. Am. Heart Assoc. 9 (5), e014397. 10.1161/JAHA.119.014397 32079474 PMC7335576

[B212] Zhang N. Aiyasiding X. Li W. j. Liao H. h. Tang Q. z. (2022). Neutrophil degranulation and myocardial infarction. Cell Commun. Signal. 20 (1), 50. 10.1186/s12964-022-00824-4 35410418 PMC8996539

[B213] Zhang Z. Shen H. Zhang S. Zhou J. Zhang M. Wang Y. (2026). Hypoxia-preconditioned cardiomyocyte-derived extracellular vesicles alleviate myocardial ischemic injury by reprogramming macrophage polarization via the Fgl2/NF-κB pathway . Int. Immunopharmacol. 170, 116077. 10.1016/j.intimp.2025.116077 41421228

[B214] Zhao Y. Sun X. Cao W. Ma J. Sun L. Qian H. (2015). Exosomes derived from human umbilical cord mesenchymal stem cells relieve acute myocardial ischemic injury. Stem Cells Int. 2015, 761643. 10.1155/2015/761643 26106430 PMC4461782

[B215] Zhao J. Li X. Hu J. Chen F. Qiao S. Sun X. (2019). Mesenchymal stromal cell-derived exosomes attenuate myocardial ischaemia-reperfusion injury through miR-182-regulated macrophage polarization. Cardiovasc. Res. 115 (7), 1205–1216. 10.1093/cvr/cvz040 30753344 PMC6529919

[B216] Zhao T. X. Aetesam-Ur-Rahman M. Sage A. P. Victor S. Kurian R. Fielding S. (2021). Rituximab in patients with acute ST-elevation myocardial infarction: an experimental medicine safety study. Cardiovasc. Res. 118 (3), 872–882. 10.1093/cvr/cvab113 PMC8859640 33783498

[B217] Zheng Z. Li Z. Xu C. Guo B. Guo P. (2019). Folate-displaying exosome mediated cytosolic delivery of siRNA avoiding endosome trapping. J. Controlled Release Official Journal Control. Release Soc. 311-312, 43–49. 10.1016/j.jconrel.2019.08.021 31446085 PMC6874920

[B218] Zheng D. Huo M. Li B. Wang W. Piao H. Wang Y. (2020). The role of exosomes and exosomal MicroRNA in cardiovascular disease. Front. Cell Dev. Biol. 8, 616161. 10.3389/fcell.2020.616161 33511124 PMC7835482

[B219] Zhu R. Sun H. Yu K. Zhong Y. Shi H. Wei Y. (2016). Interleukin‐37 and dendritic cells treated with Interleukin‐37 plus troponin I ameliorate cardiac remodeling after myocardial infarction. J. Am. Heart Assoc. Cardiovasc. Cerebrovasc. Dis. 5 (12), e004406. 10.1161/JAHA.116.004406 27919929 PMC5210436

[B220] Zhu L.-P. Tian T. Wang J. Y. He J. N. Chen T. Pan M. (2018). Hypoxia-elicited mesenchymal stem cell-derived exosomes facilitates cardiac repair through miR-125b-mediated prevention of cell death in myocardial infarction. Theranostics 8 (22), 6163–6177. 10.7150/thno.28021 30613290 PMC6299684

[B221] Zhu J. Lu K. Zhang N. Zhao Y. Ma Q. Shen J. (2018). Myocardial reparative functions of exosomes from mesenchymal stem cells are enhanced by hypoxia treatment of the cells via transferring microRNA-210 in an nSMase2-dependent way. Artif. Cells, Nanomedicine, Biotechnology 46 (8), 1659–1670. 10.1080/21691401.2017.1388249 29141446 PMC5955787

[B222] Zhu Y.-G. Shi M. m. Monsel A. Dai C. x. Dong X. Shen H. (2022). Nebulized exosomes derived from allogenic adipose tissue mesenchymal stromal cells in patients with severe COVID-19: a pilot study. Stem Cell Res. and Ther. 13 (1), 220. 10.1186/s13287-022-02900-5 35619189 PMC9135389

[B223] Zhu D. Liu S. Huang K. Wang Z. Hu S. Li J. (2022). Intrapericardial exosome therapy dampens cardiac injury via activating Foxo3. Circulation Res. 131 (10), e135–e150. 10.1161/circresaha.122.321384 36252111 PMC9667926

[B224] Zhu Y. Xi Q. Liu Y. Zhou Y. Liao J. Wu Q. (2025). Recent advances in exosome-based nanodelivery systems for parkinson's disease. Biomaterials 325, 123548. 10.1016/j.biomaterials.2025.123548 40664089

[B225] Zhuang R. Meng Q. Ma X. Shi S. Gong S. Liu J. (2022). CD4+FoxP3+CD73+ regulatory T cell promotes cardiac healing post-myocardial infarction. Theranostics 12 (6), 2707–2721. 10.7150/thno.68437 35401839 PMC8965484

[B226] Zouggari Y. Ait-Oufella H. Bonnin P. Simon T. Sage A. P. Guérin C. (2013). B lymphocytes trigger monocyte mobilization and impair heart function after acute myocardial infarction. Nat. Medicine 19 (10), 1273–1280. 10.1038/nm.3284 24037091 PMC4042928

